# Comparative assessment of SNP genotyping assays for challenging forensic samples utilizing ancient DNA methods

**DOI:** 10.1186/s13059-025-03912-z

**Published:** 2025-12-23

**Authors:** Adam Staadig, Maja Krzewińska, Maja Sidstedt, Daniel Kling, Siri Aili Fagerholm, Ricky Ansell, Anders Götherström, Andreas Tillmar

**Affiliations:** 1 Department of Forensic Genetics and Forensic Toxicology, National Board of Forensic Medicine, Artillerigatan 12, Linköping, SE-587 58 Sweden; 2 Department of Biomedical and Clinical Sciences, Faculty of Health Sciences, Linköping University, Linköping, SE-581 83 Sweden; 3 Centre for Palaeogenetics, Svante Arrhenius väg 20C, Stockholm, SE-106 91 Sweden; 4 Department of Archaeology and Classical Studies, Stockholm University, Wallenberglaboratoriet, Lilla Frescativägen 7, Stockholm, SE-114 18 Sweden; 5 National Forensic Centre, Swedish Police Authority, Linköping, SE-581 94 Sweden; 6 Department of Forensic Sciences, Oslo University Hospital, Oslo, Norway; 7 Department of Physics, Chemistry and Biology, Linköping University, Linköping, SE-581 83 Sweden

**Keywords:** Forensic genetics, Ancient DNA, Degraded DNA, Forensic investigative genetic genealogy, SNP, Bone, Hybridization capture, Whole-genome sequencing, Massively parallel sequencing

## Abstract

**Background:**

The fields of ancient DNA research and forensic genetics share both methodological similarities and common challenges, particularly in the analysis of degraded DNA. Leveraging these overlaps, this study evaluates three single nucleotide polymorphisms (SNP)-based genotyping assays for analyzing challenging forensic samples: the FORCE-QIAseq SNP panel, the Twist ancient DNA hybridization capture panel, and whole-genome sequencing.

**Results:**

We analyze twenty skeletal bone and tooth samples from authentic missing person cases, where almost all samples are severely degraded and contain exceptionally low amounts of endogenous DNA, reflected by both reduced quantifiable DNA concentrations and lower proportions of human DNA reads than typically obtained from high-quality forensic samples. Despite these challenging sample characteristics, both the FORCE and Twist assays successfully generate a substantial number of genotypes across many samples, while whole-genome sequencing yields fewer SNP calls. However, techniques like probabilistic genotyping, increase sequencing depth or genotype imputation can further enhance the utility of WGS for forensic use.

**Conclusions:**

This study highlights the effectiveness of incorporating ancient DNA methods into forensic genetics for the analysis of degraded samples. The findings are broadly applicable to both forensic and ancient DNA research disciplines, offering valuable insights into assay selection based on sample condition and investigative goals.

**Supplementary Information:**

The online version contains supplementary material available at 10.1186/s13059-025-03912-z.

## Background

Forensic genetics is a discipline that employs DNA analysis to yield scientifically valid evidence admissible in legal contexts. This encompasses matching perpetrators with crime scene evidence, identifying human remains, and assessing kinships. Standard forensic DNA analysis involves DNA extraction from various specimens, polymerase chain reaction (PCR) amplification of short tandem repeat (STR) markers, and subsequent detection of DNA fragments using capillary electrophoresis. DNA samples from a reference individual, such as a suspect or a presumed relative of the unknown, are included in statistical calculations to determine the evidential weight of the results, providing statistical likelihood of sample and reference DNA match [1]. Standard forensic DNA typing is reliant on relatively large intact DNA fragments, due to the fragment size of the STRs (up to several hundred base pairs) [2]. This conventional procedure has been widely applied for decades and is both labor-efficient and cost-effective. However, the quality of forensic samples is heavily influenced by factors such as the time since deposition, environmental conditions, and postmortem treatment of remains often leading to e.g. enzymatic inhibition [3] and DNA degradation [4]. These mechanisms can disturb the amplification process and compromise DNA typing success. Consequently, there has been a growing emphasis on utilizing single nucleotide polymorphisms (SNPs) and massively parallel sequencing (MPS) in forensic analyses to address the limitations associated with current STR typing technologies [5–9]. While individual SNP variants offer less discriminatory power compared to STRs, due to their typically limited allelic variation, recent technological advancements have facilitated the simultaneous multiplexing of thousands to millions of SNPs [10–14]. Furthermore, the limited number of traditional STR markers may result in reduced informativeness, particularly in cases requiring the inference of distant relationships. In such instances, multiplex SNP analysis has demonstrated significant discriminatory power, enhancing the evidential weight of the forensic investigation [10, 14, 15]. Moreover, MPS analysis of SNPs has enabled the prediction of an unknown’s biogeographical ancestry [16] and phenotype, such as eye-, hair- and skin color [17] from DNA. These types of investigative leads can assist identification efforts and lead the investigation forward.

Another approach to investigative leads that has recently gained a lot of attention is forensic investigative genetic genealogy (FIGG), known by numerous names [18], which has been adopted as a powerful tool by the forensic community [19]. This approach takes advantage of the fact that hundreds of thousands of people have uploaded their DNA in law-enforcement accessible commercial genealogical databases [20] and that biological relatives share segments of DNA. The method has been applied by law enforcement, mainly in the US, both when identifying human remains as well as solving hundreds of murders and other violent crimes [21]. Moreover, several cases in Europe [22–25] and Australia [26, 27] have either already employed or are poised to utilize FIGG for identifying missing persons and perpetrators. An extended DNA profile is required for successful use of the FIGG method, ideally comprising hundreds of thousands of SNPs, to find the distant relatives [28]. Currently, mainly whole-genome sequencing and microarray genotyping have been applied for obtaining these high-density SNP profiles [28]. However, smaller panels like the ForenSeq Kintelligence kit (Qiagen, Hilden, Germany) which targets approximately 10 000 SNPs, have also proven effective in a selection of cases [11, 13, 29]. A forensically validated method is often used to either confirm or reject the potential candidate identified via FIGG. This is typically done via STR typing, however, due to the aforementioned limitations of STRs, a forensically validated SNP based approach is beneficial when depending on degraded samples or distant kinships. One such SNP assay developed for forensic usage is the FORCE panel [10], comprising approximately 5 500 SNPs.

One of the greatest challenges in forensic DNA analysis is the frequent occurrence of samples with both low quality and low quantity. Notably, many challenges in forensic genetics overlap with those faced in the closely related field of ancient DNA (aDNA) research, where minimal and degraded DNA are common obstacles. In recent years, there has been an increased interest in sharing knowledge between these disciplines due to their many similarities [30–34]. The field of aDNA research has made significant improvements by embracing innovative technologies. The introduction of high throughput sequencing has led to numerous breakthroughs in understanding human evolution, from sequencing the first ancient human genome [35] to uncovering genetic insights of other hominins [36, 37]. More recently, aDNA researchers have successfully recovered million-year-old DNA from both a mammoth tooth [38] and permafrost sediment [39]. Nonetheless, DNA degradation remains an inevitable challenge encountered in aDNA studies [40–42]. Numerous technological advancements have focused on optimizing the recovery of damaged DNA, including improvements in DNA extraction methods [43–46] and hybridization capture-based target enrichment technologies [30, 46–49].

The primary technology for target enrichment in forensic genetics is PCR-based amplicon enrichment [30]. Typically, two target-specific primers per site are used to amplify the region of interest. In contrast, the most common enrichment method in ancient DNA research is hybridization capture technology [30]. This method utilizes oligonucleotide probes, or baits, which hybridize to specific targets of interest which further enables simultaneous analysis of millions of SNPs up to the entire exome. These enrichment strategies typically utilize different library preparation methods. PCR-based panels often use amplicon-based workflows, which integrates primer tagging and indexing during amplification. Hybridization capture, on the other hand, usually commence with DNA fragmentation, following with blunt-end repair and adapter ligation, and can be performed using either double stranded or single stranded DNA library preparation methods [50]. The use of hybridization capture technologies in routine forensic case work is limited, nevertheless, several forensic research projects covering the method has recently been published [14, 15, 51–54].

In ancient DNA research, the so called, 1240 K reagent SNP panel [55–57] has been widely utilized in recent years [58]. These SNPs were specifically selected to investigate genetic variation among modern human populations. Recently, Twist Biosciences, in collaboration with the David Reich lab at Harvard University developed a new SNP panel: the Twist ancient DNA panel [58]. This panel comprises the 1240 K SNPs along with additional phenotype-informative and Y-SNPs, targeting a total of 1,352,259 SNPs. Thus, most of these SNPs are relevant to the forensic community, including information relating to biogeographic ancestry, phenotype, and kinship.

In the current study, the aim was to integrate best practices from ancient DNA methods into forensic genetics and explore the potential advantages of incorporating these procedures to enhance the outcomes of forensic investigations. We have evaluated and compared standard STR typing with three different SNP genotyping assays. With the findings from this study, we aim to aid the decision making when selecting SNP assays, not only for cases employing FIGG but also for broader forensic investigations, including the identification of human remains, kinship inference and the identification of individuals from crime scene evidence.

## Results

**Fig. 1:**
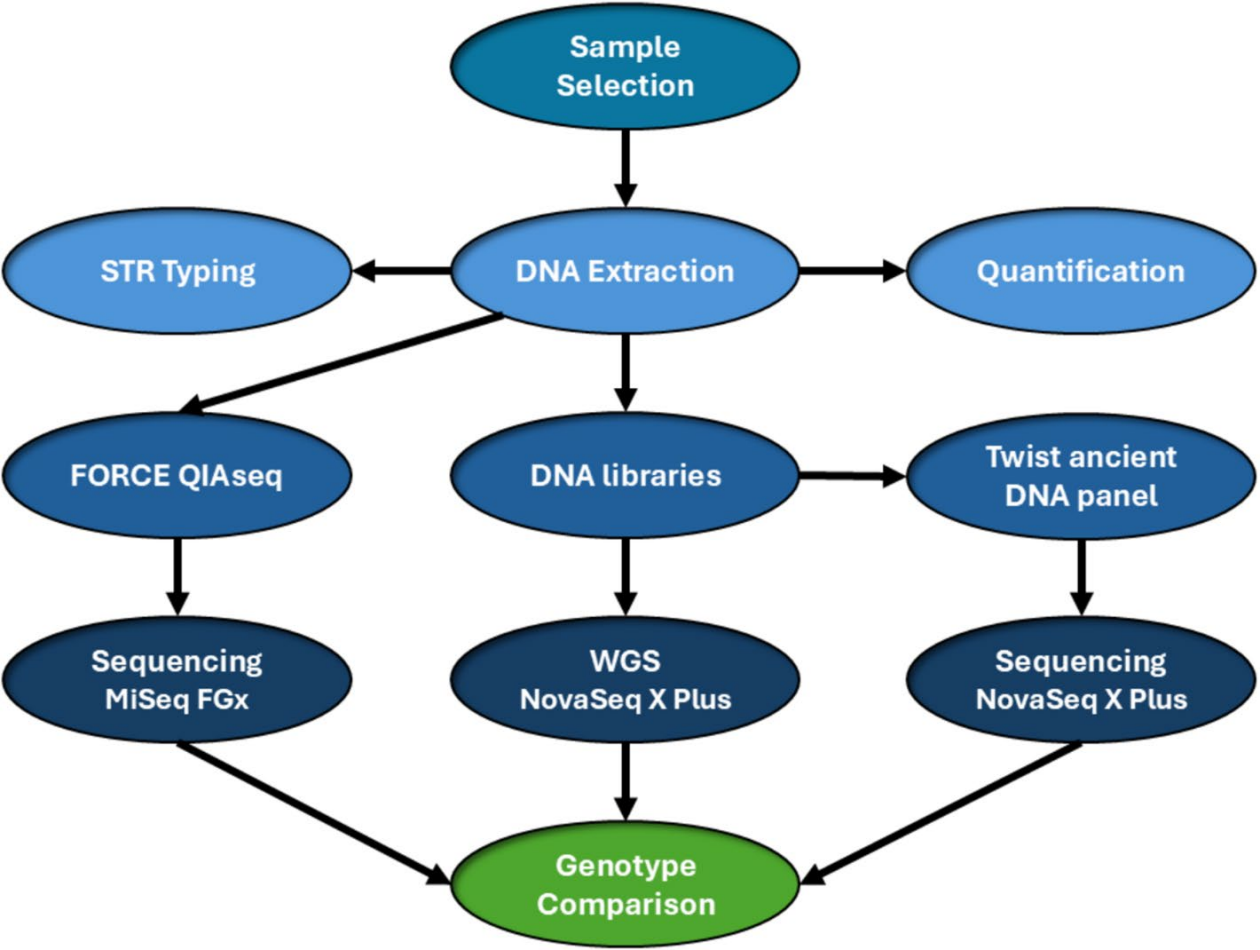
A schematic overview of the methodology used in this study. DNA from selected bone and tooth samples was extracted and quantified. STR analysis was performed, and three different SNP genotyping assays were evaluated

In this study, DNA was extracted from twenty human skeletal bone and tooth samples of presumed modern origin (< 30 years old), using a silica-based spin-column method applied to approximately 50 mg of bone powder. Standard forensic STR typing by capillary electrophoresis (CE) was performed and compared with three SNP typing assays. These included two targeted assays, the FORCE QIAseq panel (Qiagen) and the Twist aDNA panel (Twist Biosciences, San Francisco, CA, USA), as well as whole-genome sequencing (WGS). An overview of the methodological workflow is presented in Fig. 1, with full experimental details provided in Sect. 5 (Methods).

DNA quantification of the extracted DNA was performed using both the PowerQuant system and the Qubit 2.0 fluorometer. All samples demonstrated low DNA amounts, with all except two displaying concentrations below 0.06 ng/µl as measured by PowerQuant (Table 1). DNA concentrations measured by Qubit were consistently higher than those measured with PowerQuant, ranging from < 0.005 ng/µl (below the detection limit) to 8.7 ng/µl (Table 1). For context, 0.5–2 ng DNA is typically required in commercial forensic STR kits, however, lower input is common for forensic bone samples [2]. Additionally, degradation index for available samples were > 2, indicating DNA degradation in those samples (Table 1). Furthermore, the quality of the samples was assessed by interpreting the fragment length distribution of mapped reads and postmortem damage plots from the WGS analysis (Additional file 1: Figures S1 and S2).

**Table 1:** The type of bone sample selected for analysis and DNA quantification results measured with Qubit and PowerQuant qPCR. Additionally, the degradation index obtained from PowerQuant is shown for those samples with a successful quantification of both the long and the short amplicon. NA indicates that no value could be calculated. Samples with concentrations below the detection limit failed amplification of both amplicons, while the remaining failed the long amplicon

Sample name	Material analyzed	Qubit DNA Concentration (ng/µl)	PowerQuant qPCR DNA Concentration (ng/µl)	PowerQuant Degradation index
001	Tibia bone	0.141	< 0.0005	NA
002	Petrous temporal bone	0.43	< 0.0005	NA
003	Cranium	0.53	0.0025	6.25
004	Bone unspecified	0.296	0.0010	NA
005	Humerus bone	0.176	0.0040	NA
006	Bone unspecified	< 0.005	< 0.0005	NA
007	Cranium	8.7	0.0005	NA
008	Cranium	5.19	< 0.0005	NA
009	Bone unspecified	0.269	0.0078	NA
010	Cranium	2.73	< 0.0005	NA
011	Rib bones	3.14	1.3322	5.17
012	Femur bone	2.96	0.0028	NA
013	Tooth	1.5	< 0.0005	NA
014	Cranium	0.352	0.0024	4
015	Bone unspecified	0.429	0.0009	NA
016	Tooth	1.36	0.1038	NA
017	Femur bone	0.444	0.0014	4.67
018	Femur bone	0.109	0.0183	20.33
019	Bone unspecified	0.117	0.0600	20.69
020	Femur bone	0.88	0.0171	34.2

**Fig. 2:**
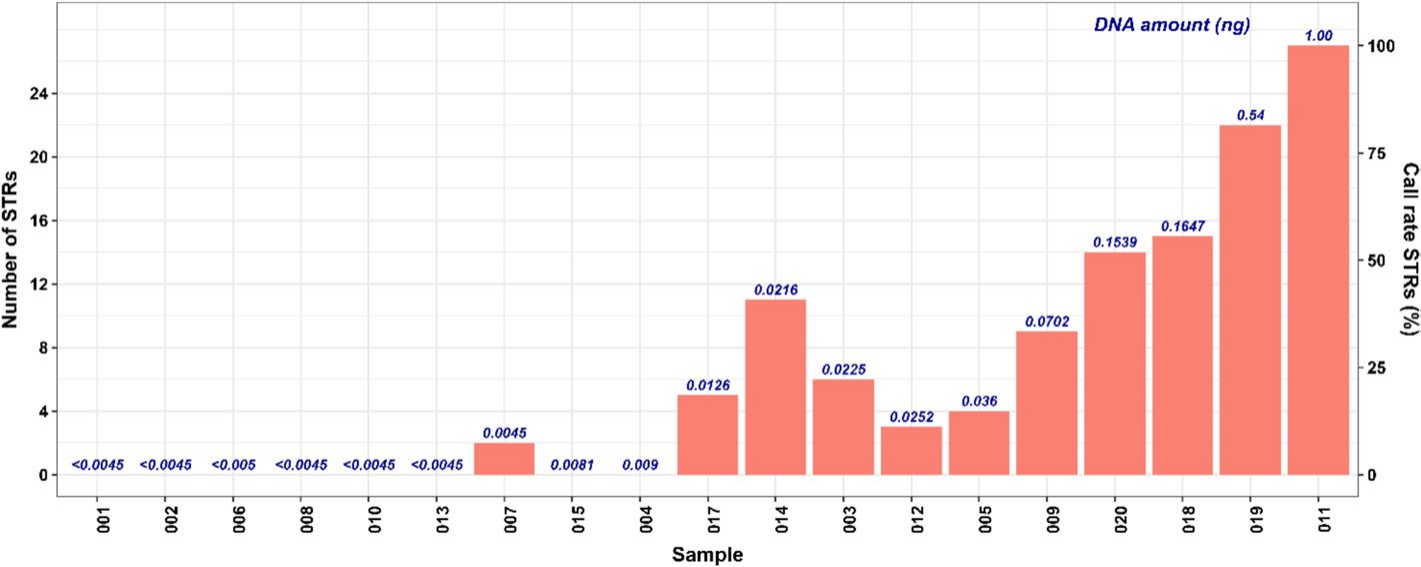
Number of STR markers with alleles above the analytical threshold (left Y-axis) and the call rate (right Y-axis). The blue values above each bar represent the total amount of input DNA (ng). Note that sample 016 was not analyzed due to the limited amount of DNA extract

Capillary electrophoresis based STR analysis was conducted for nineteen of the samples (sample 016 was excluded due to limited amount of DNA extract), the positive- and negative controls using the PowerPlex Fusion 6C system. Complete STR profiles were observed for the two positive controls and no alleles were typed for the negative controls. Specific thresholds for typing an allele had previously been established from internal validation, a common process where laboratories define method-specific analytical thresholds based on controlled testing to demonstrate that a method is fit for the intended purpose. In forensic laboratories, such validation is often required to ensure that only reliable signals are considered in casework. Signals above the in-house validated analytical thresholds (RFU) were detected for eleven samples, yielding between 2 and 27 STR markers with an allele call (Fig. 2). The input DNA amount was relatively low for all samples (Fig. 2), considering that the optimal input for CE-based STR kits is approximately 0.5–2 ng of human DNA [2]. This low DNA amount was mainly due to a combination of limited DNA yield and DNA degradation (Table 1). Due to sample limitations, a maximum of 10 µl DNA extract was added and the reaction was adjusted to the required 15 µl PCR volume with water (Additional file 2: Table S1). Since the protocol allows for up to 15 µl DNA, this lower input may have influenced the STR typing results.

**Fig. 3:**
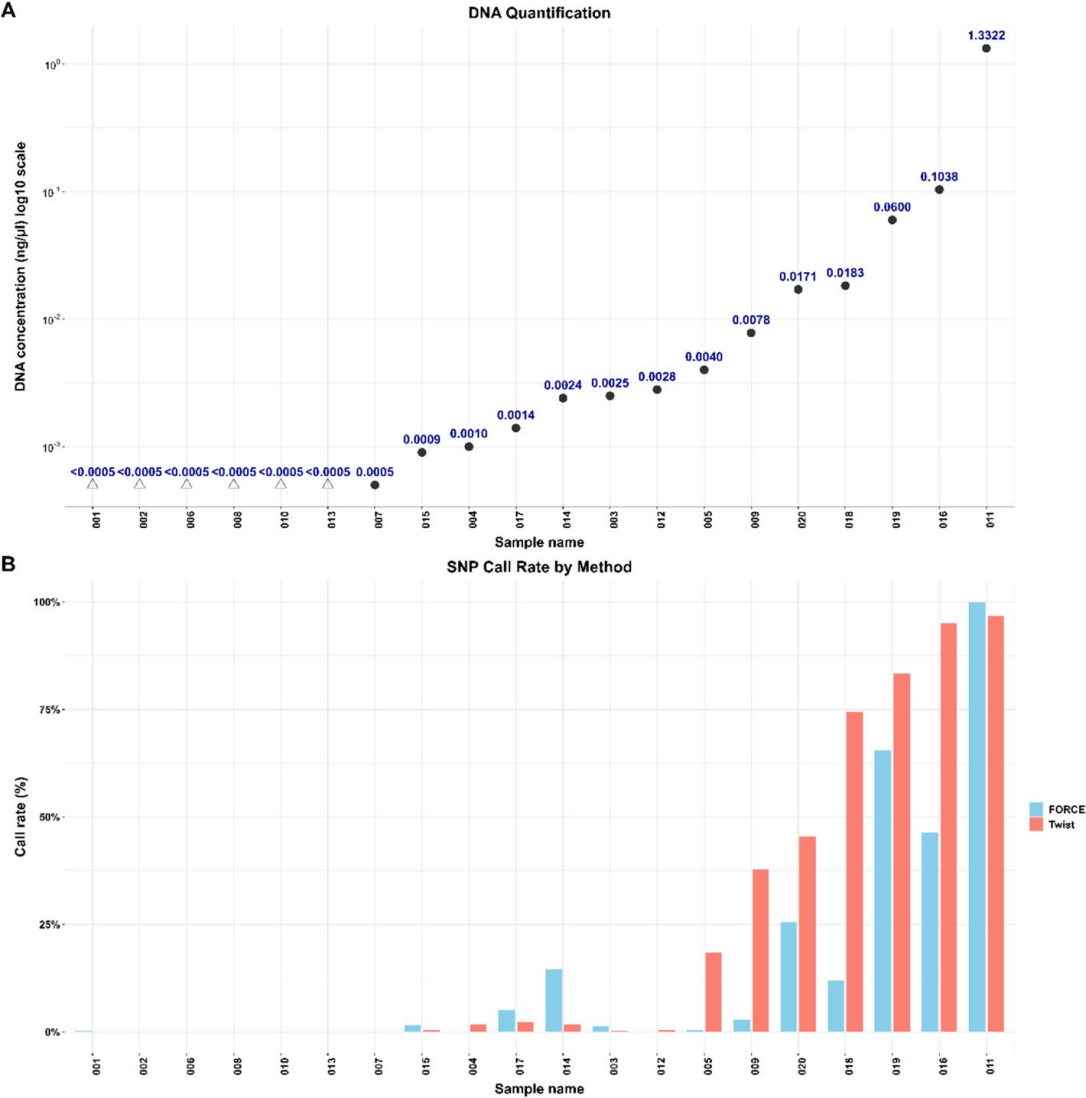
**A** DNA concentration for all samples measured with PowerQuant illustrated as a dot plot with a logarithmic scale. Triangles represent samples below the detection limit of the assay (0.0005 ng/µl), and the value at each dot represents the observed DNA concentration in ng/µl. **B** the proportion of called SNPs per sample for the FORCE assay (blue), and the Twist assay (red)

We further evaluated the performance of the extracted DNA using the FORCE panel, a forensically validated MPS-based SNP assay. In total, 5,379 SNPs were analyzed, selected based on previous publications [10, 59] and internal validation. A list of the selected markers can be found in Additional file 2: Table S2. Sequencing reads were bioinformatically analyzed in CLC Genomics Workbench (Additional file 1: Figure S3) and subsequent SNP genotyping was performed according to assay-specific thresholds (Additional file 2: Table S3), as detailed in Methods Section 5.9. The total number of typed FORCE SNPs per sample is illustrated in Fig. 3. Ten samples yielded more than 60 typed SNPs, six samples had over 500 SNPs typed, and one sample (011) had nearly all genotypes called. The call rate for the positive controls was > 99.9% for both samples, with 5,376 SNPs for NA12877 and 4,512 SNPs for NA12878 passing the calling thresholds. The difference in SNP counts reflects the assay design, as Y-SNPs are included. As a result, the total number of targetable SNPs was 5,379 for the male control sample (NA12877) and 4,513 for the female control (NA12878). None of the three extraction blanks had any genotypes passing the criteria for typing. The exact number of called SNPs per sample (with and without Y-SNPs) is detailed in Additional file 2: Table S4.

The Twist aDNA panel targets 1,352,259 SNPs, details of the complete marker set can be found in [58]. The bioinformatic workflow is illustrated in Additional file 1: Figure S4 and the SNP calling thresholds are presented in Additional file 2: Table S5, for details see Methods Section 5.9. The number of called SNPs ranged from 25 (0.002%) to 1,309,327 (96.8%) and more than 800 SNPs were observed in 16 of the samples (Fig. 3). The two positive controls achieved call rates exceeding 99% with NA12877 and NA12878 having 1,339,280 and 1,262,047 called SNPs, respectively. For the three negative controls, 350 (0.03%), 220 (0.02%) and 102 (0.007%) SNPs passed the typing thresholds. The exact number of called SNPs per sample (with and without Y-SNPs) is listed in Additional file 2: Table S6.

Evaluation of the WGS analysis was conducted using the same set of 1.3 million SNPs from the Twist aDNA panel for comparison. The WGS libraries were sequenced across three different sequencing runs, and the resulting FASTQ files were merged prior to genotype calling. However, across all samples, only a limited number of genotypes met the typing thresholds. The highest number of typed SNPs was observed in sample 019 which had 448 SNPs called (0.03%). The low call rate was caused by low average coverage per SNP, which was 0.3 × on average across all samples for the Twist targets. The number of called SNPs per sample is specified in Additional file 2: Table S6. However, SNPs across the complete genome should be considered to estimate the true potential of WGS analysis. Therefore, we calculated the number of called genotypes for sample 019 at 29,083,171 SNP sites across all autosomes and the X-chromosome. Among these SNPs, 18,347 genotypes (0.06%) passed the typing thresholds. As described in Sect. 2.3, eight samples were re-extracted. Data presented here is based on merged sequencing reads from all DNA libraries, including those built from re-extracted DNA. Preliminary analysis of the separate DNA extraction methods showed, in general, that none of the examined extraction methods outperformed the other (data not shown).

**Fig. 4:**
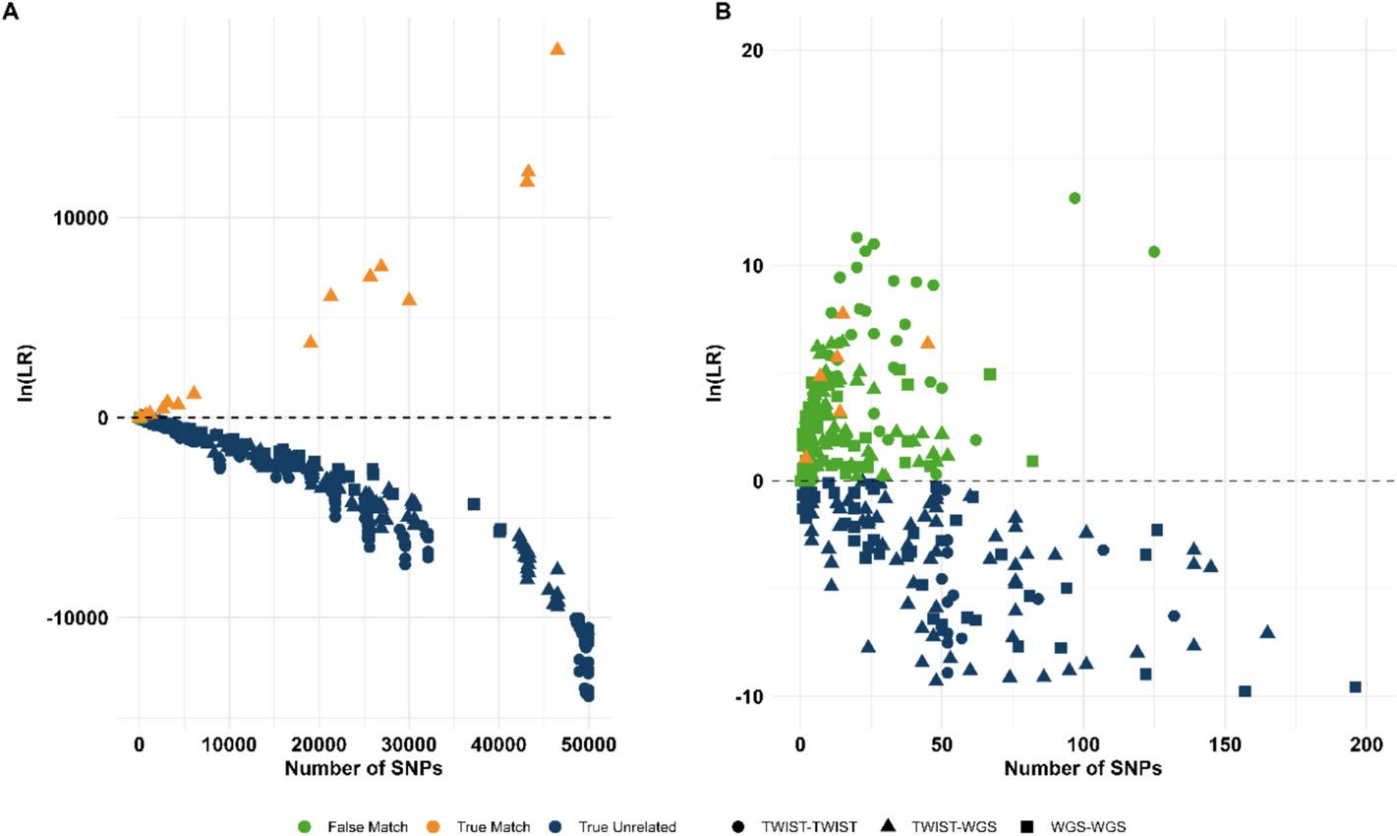
A dot plot with log-likelihood ratio values (natural logarithm, ln(LR)) of direct match versus unrelated for all sample pairs (Twist-Twist as circles, Twist-WGS as triangles and WGS-WGS as squares) and the number of typed SNPs is illustrated. The true related sample pairs are shown in orange, the true unrelated in dark blue and the false matches in green. **A** shows all samples and **B** emphasizes the sample comparisons with ln(LR) values close to zero, highlighting the false positive comparisons (green)

We utilized likelihood ratio (LR) calculations of the hypotheses *Direct match* versus *Unrelated* to estimate the concordance of the Twist and WGS generated genotypes. This was performed pairwise between all samples, including the two positive controls, using FamLink2 [60]. Genotypes were probabilistically assigned in the software based on observed sequencing read count. Twenty-two sample comparisons represent true direct matches (comparing genotypes from Twist and WGS for the same sample), while the remaining 946 comparisons are true unrelated pairs, assuming that the skeletal remains are from unrelated individuals. The LR for each comparison is illustrated as a dot plot in Fig. 4. All true direct matches showed positive log-likelihood ratio values (natural logarithm, ln(LR)) ranging from 1 to 18,370, with a median of 536. Most unrelated sample pairs displayed a negative ln(LR), interpreted as a true negative, although 166 unrelated sample pairs showed a ln(LR) > 0, thus interpreted as false positives. The maximum number of typed SNPs for these false positives was 125, with a median of 9 SNPs. The median ln(LR) for the false positives was two, reaching a maximum of 13. Notably, sample pairs with the highest LR among false matches were observed in comparisons where both samples originated from Twist-generated data, indicating a potential bias effect within the Twist analysis.

Genotype accuracy was further assessed by comparing observed FORCE, Twist and WGS genotypes with genotypes obtained from previously published high-depth FASTQ files for NA12877 and NA12878. Concordance rates and the number of typed SNPs in each data set are presented in Table 2, while discordant SNPs are listed in Additional file 2: Table S7. Most of the errors (80%) were false homozygotes. Further investigation including replicate analysis and additional control samples are required to determine the cause of these few discordant SNPs.

**Table 2:** The genotype accuracy for each of the three methods; FORCE, Twist and WGS, are shown together with the total number of typed SNPs

Sample	FORCE		Twist		WGS	
	Genotype accuracy (%)	Typed SNPs	Genotype accuracy (%)	Typed SNPs	Genotype accuracy (%)	Typed SNPs
NA12877	100%	5,376	99.992%	1,330,214	99.193%	124
NA12878*	100%	4,512	99.995%	1,253,735	96.175%	183

^*^Excluding Y-SNPs

**Fig. 5:**
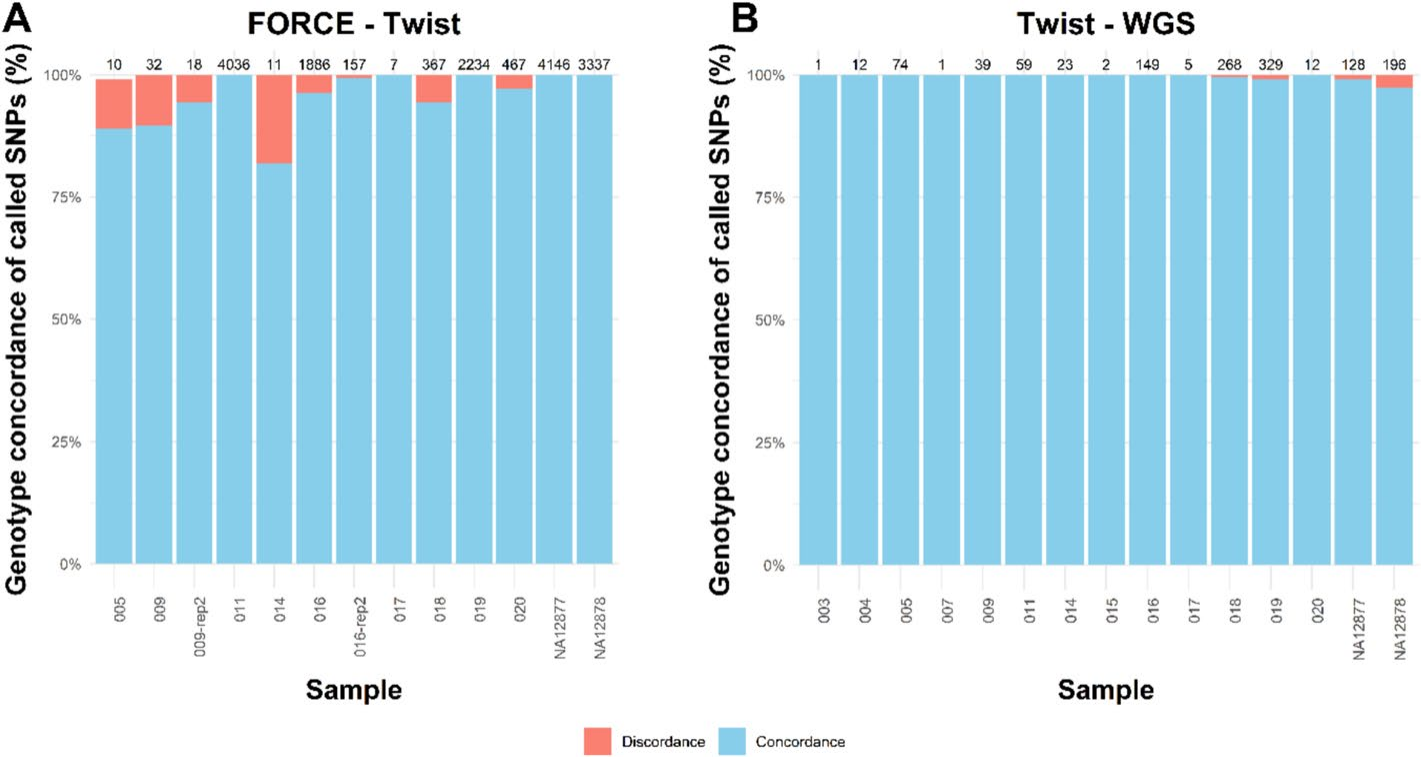
Genotype concordance (blue) and discordance (red) for samples with overlapping typed genotypes. The number above each bar represents the number of overlapping typed SNPs in the comparison. Sample 009 was analyzed in replicate with the Twist assay, labeled as 009-rep2 in the figure. Sample 016 was analyzed in replicate with the FORCE assay, labeled as 016-rep2. **A** shows concordance between FORCE and Twist, and **B** represents the Twist and WGS comparisons

Genotype concordance was assessed between each pair of SNP assay. The theoretical maximum number of overlapping SNPs between the FORCE panel and the Twist aDNA panel was 4,175. Concordance rates and the total number of overlapping typed SNPs between FORCE and Twist are illustrated in Fig. 5A for eleven comparisons, including the positive controls. The remaining eleven samples had no overlapping typed SNPs between these assays. Complete genotype concordance for typed SNPs was observed for the positive controls and two samples (011 and 017). For the other seven samples, concordance rates ranged from 81.8% to 99.9% with discordant SNPs ranging between 1 and 21 except for one sample (016) which displayed 72 discordant genotypes. Notably, sample 016 was analyzed in duplicate using the FORCE assay. To increase the reliability of the genotypes, only SNPs that generated reproducible results in both FORCE replicates were retained for comparison. When this consensus genotype set from FORCE was compared with Twist genotypes, only a single SNP remained discordant among 157 overlapping genotypes. This shows that combining reproducible genotypes from replicate analyses can reduce the number of discordances, although the total number of overlapping SNPs may decrease, the concordance rate improves due to the increased reliability in the observed genotype. Only 0.5% of all compared genotypes (119 out of 25,014) were discordant, indicating high overall concordance. One discordant genotype was a contradictory homozygous call, while the rest were either heterozygous or homozygous across the assays, indicating potential drop-in of a false allele or drop-out of a true variant. Additionally, two DNA libraries originating from the same sample (009) were sequenced with Twist. When comparing each Twist replicate individually with FORCE, up to four discordant SNPs were observed. However, when only SNPs that yielded concordant genotypes across both Twist replicates were retained for comparison, the number of discordant SNPs decreased to one. To further assess concordance, we measured pairwise concordance rates between all nine samples to determine the consistency of genotypes across libraries from different individuals. As expected, samples from different individuals had substantially lower concordance rates compared to those from the same individual, with an average concordance rate of 44% for different individuals and 94% for the same individual. The concordance rate and the number of compared SNPs for each sample pair are illustrated in Additional file 1: Figure S5.

Genotype concordance was also measured between Twist and WGS data for 15 samples that had at least one overlapping SNP call, and of those, 11 samples showed complete genotype concordance (Fig. 5). The remaining samples had no overlapping SNPs between the assays. Similar to the FORCE-Twist comparison, we evaluated genotype concordance across all sample pairs (Additional file 1: Figure S6). Results showed an unexpectedly high concordance rate among samples from different individuals, likely due to the limited number of overlapping genotypes and the fact that nearly all of these SNPs were Y-chromosomal. These Y-SNPs have lower discriminatory power compared to the autosomal SNPs, which likely contributed to the increased concordance rates. Since Y-SNPs can inflate concordance, we repeated the concordance analyses by excluding them (Additional file 1: Figure S7). For FORCE-Twist, concordance rates were mainly unchanged since many autosomal SNPs overlapped. For Twist-WGS, excluding Y-SNPs removed some overlapping SNPs in several samples, leaving only very few autosomal SNPs in total, but concordance remained high where overlap existed (97–100%). No typed SNPs were shared between the FORCE and WGS datasets, wherefore genotype comparisons across all three assays could not be performed.

As previously mentioned, one sample (016) was analyzed in two replicates using the FORCE assay. Of the 222 overlapping genotypes that met the typing thresholds, 195 (87.8%) were concordant. The total call rate varied significantly between the replicates, 5% and 46%, respectively. In the Twist assay, four samples (009, 010, 011 and 012) were analyzed in replicates, both with and without initial fragmentation step. Sample 010 exhibited complete concordance, although only nine genotypes overlapped. Sample 011 yielded over 1.29 million typed SNPs, achieving a genotype concordance rate exceeding 99.99% with just 14 discordant genotypes. For sample 009, the number of compared genotypes was 203,294, resulting in a concordance rate of 98.8% and 2,432 discordant genotypes. Finally, sample 012 displayed a genotype concordance rate of 95.6% with twelve discordant genotypes identified among 272 overlapping typed SNPs.

A multivariate linear regression analysis was performed to evaluate the impact of various sequencing parameters on SNP calling. The parameters analyzed included DNA input concentration, total sequences, trimmed sequences, human reads filtered with the *Taxonomic profiling* tool, reads mapping to the human reference genome, reads mapping on-target, and PCR duplicates (Additional file 2: Tables S4 and S6). In the FORCE data set, reads mapping to the human genome (*p* = 0.0135) was the only significant factor influencing the number of called SNPs. Both the human reads filtered with the *Taxonomic profiling* tool (*p* = 0.0006) and PCR duplicates (*p* = 0.006) had strong impact on SNP calling in the Twist data set. In the WGS data set, *Taxonomic profiling* reads had a highly significant effect on SNP calling (*p* < 0.001), with DNA input concentration also showing a significant influence (*p* = 0.007). *P*-values from the analysis for all included parameters are presented in Additional file 2: Table S8.

**Fig. 6:**
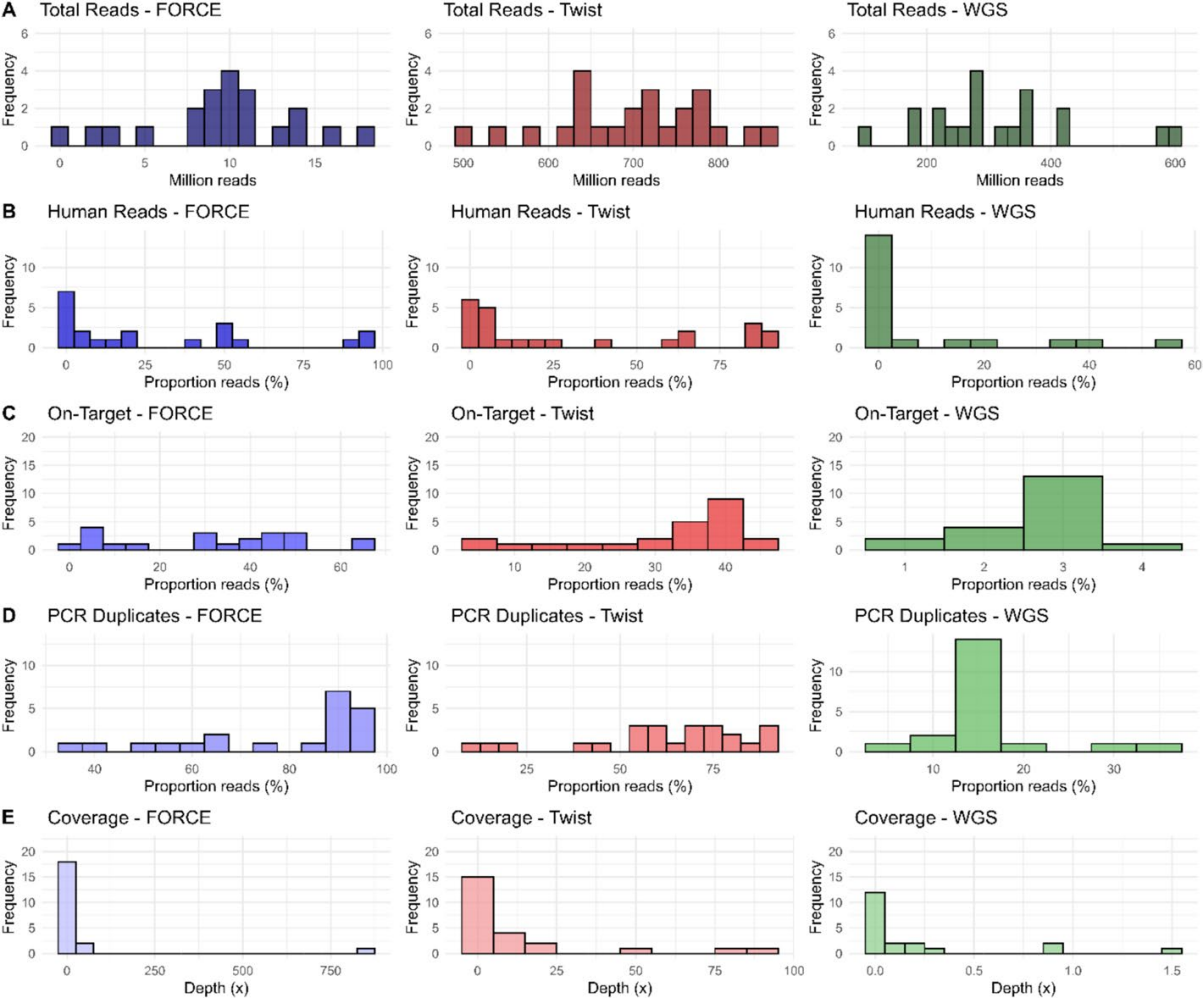
Distribution of different parameters across all samples for the three SNP assays; FORCE (blue), Twist (red), and WGS (green). **A** shows number of total reads, **B** proportion of human reads, **C** proportion of reads on-target, **D** proportion PCR duplicates, and **E** coverage

The distribution of various sequencing parameters for the three SNP assays across all samples is illustrated in Fig. 6, and detailed in Additional file 2: Table S4 (FORCE) and Table S6 (Twist and WGS). As expected, the variation in total reads (Fig. 6A) primarily reflects the difference in theoretical yield of the sequencing platforms and pooling conditions rather than assay specific differences. The proportion human reads (Fig. 6B) was calculated as mapped reads to the human reference genome for FORCE and from *Taxonomic profiling* for Twist and WGS to enable comparability. The proportion of on-target reads (Fig. 6C) illustrates substantial variation in FORCE, whereas most Twist samples averaged around 30%. Additionally, when extending the target region with 100 bp downstream and upstream of the SNP, the proportion on-target mapping increased on average to 40% (Additional file 2: Table S6). This indicates that the remaining 60% align completely off-target. The low proportion on-target reads for WGS is expected since only Twist SNPs were used as reference targets, while WGS covers the whole genome. The PCR duplication levels (Fig. 6D) were similar for FORCE and Twist, whereas WGS displayed relatively few duplicates. While the overall coverage distribution (Fig. 6E) was similar across assays, with most samples yielding low read depths and a few outliers with higher coverage, the range of coverage differed substantially, with WGS showing the lowest absolute values.

Performance comparisons among the investigated assays (STR, FORCE, Twist, and WGS) were evaluated based on the call rate per sample for each method, which was calculated by dividing the number of observed markers with the total number of markers in each assay. First, a one-way ANOVA was performed across all twenty samples, revealing significant difference in the average call rate between assays (*p* = 0.0265). Tukey’s post-hoc test was then applied for pairwise comparisons showing that WGS had a significantly lower call rate than both STR (*p* = 0.042) and Twist (*p* = 0.043). No statistically significant differences were observed for any of the other comparisons. Furthermore, a one-way ANOVA conducted on STR, FORCE, Twist, and the call rate of the 5000 dynamically called WGS SNPs showed a statistically significant difference (*p* = 0.0395). However, Tukey’s post-hoc test revealed no statistically significant differences between any of the assays in this analysis. *P*-values for all comparisons are provided in Additional file 2: Table S9. Although call rates appeared similar across assays, the absolute number of usable SNPs differed substantially, which may be a more informative measure of assay performance.

## Discussion

In this study, different SNP genotyping assays were explored using twenty challenging bone and tooth samples from unidentified human remains derived from unsolved cases. While the SNP genotyping results presented are promising, given the challenging nature of these samples, several potential improvements could be explored to further enhance the performance, which are discussed in the following paragraphs.

Exceptionally low amounts of DNA were observed for most samples, highlighting that these samples are not typical standard forensic bone samples, but rather represent challenging cases at the lower end of DNA quantity and quality. Moreover, the observed damage patterns (Additional file 1: Figure S2) were, for most samples, consistent with those typically encountered in ancient DNA samples, further emphasizing the low quality of these samples. The quantification results differed between Qubit and PowerQuant, which is expected and aligns with previous studies [61, 62], as the methods employ different measurement principles. The PowerQuant assay specifically measures human DNA through real-time PCR, while Qubit uses a fluorescent dye that binds to all present DNA. Therefore, the observed difference may suggest bacterial contamination, which is likely, given the characteristic and origin of the samples. According to the authors' current in-house quantity thresholds, 13 of the samples would not have been eligible for subsequent STR analysis due to the low DNA concentration (below 0.001 ng/µl) obtained from the qPCR measurement. However, these samples may still be suitable for MPS-based analysis. Furthermore, the degradation index for all samples indicated severe DNA degradation, which was also apparent in the obtained electropherograms where the larger peaks were undetected in many samples (data not shown). Additional analyses to assess the quality of the samples were excluded due to the limited amount of extracted DNA. However, understanding the fragmentation and the proportions of single stranded and double stranded DNA is beneficial, since these factors can influence the success of subsequent library preparation, as discussed in [50, 63].

For most samples, only few STRs passed the analytical thresholds, and the informativeness from such few markers is quite limited. Only three samples yielded more than 15 STRs above the analytical threshold. Although only a limited volume of extracted DNA was used in the STR analysis, increasing the input volume would likely have only marginally improved the call rate, as the DNA concentrations were very low, and developmental validation data indicate that similar low concentration samples generally yield few typed markers [64]. In contrast, the FORCE analysis showed that ten samples had more than 60 called SNPs which is approximately equivalent to 15 STRs [65, 66]. However, this equivalence is influenced by several factors, including the specific population, mutations and type of SNP markers used. With this assumption, the results from the FORCE analysis are likely to be more informative than relying solely on the STR data. Additionally, the selected SNPs provide not only identity and kinship information, like the STRs, but also phenotype and ancestry information which can be valuable when attempting to identify unknown sample contributors. However, accurate predictions require that a sufficient number of and specific SNPs are genotyped, for example missing even a few HIrisPlex-S markers can prevent phenotype prediction. The Twist analysis yielded even more genetic information, with over 800 typed SNPs for 16 of the samples. While the results from STR, FORCE and Twist are somewhat similar, as the same sample performed consistently across the assays, the utility of an observed result may vary due to the absolute number and type of markers. As mentioned in Sect. 2.7, the DNA library concentration was limited, and further optimizations affecting library protocol selection should be considered. Further improvements could potentially be achieved utilizing single stranded library preparation protocols [67]. Additionally, preliminary results from a Twist library preparation protocol [68] suggest that enhancements regarding DNA library yield can be achieved (data not shown). Similarly, alternative library preparation strategies that intentionally overamplify to reach recommended input amounts have also proven effective, as demonstrated in a study applying the same Twist panel to forensic bone samples, which reported slightly higher SNP recovery compared to our results [69]. Furthermore, Zedda et al. [70] yielded successful results with the Twist panel when applying a protocol described in [71] on ancient DNA samples. Further optimization of the bioinformatic workflow may slightly increase the proportion of useable reads, thereby improving call rate. One such alternative is to bypass the *Taxonomic profiling* step and directly perform read mapping, which is more time-consuming but practical when processing only a limited number of samples.

The WGS data exhibited overall low average coverage per SNP, 0.3x, while the theoretical value is 26x. However, based on observed proportion of human reads and levels of PCR duplicates (Additional file 2: Table S6), the decreased coverage is somewhat expected. Consequently, considerably fewer genotypes were called in the WGS data using standard forensic SNP typing approaches, which rely on specific read thresholds for determining genotypes. In contrast, we applied an alternative approach based on probabilistic genotyping. Rather than assigning alleles prior to calculating the likelihood ratios (LR), we examined the method described in [72] using the FamLink2 software. The LR was calculated based on the observed sequencing read depth for each allele instead of predetermined genotypes. This strategy performed well, as illustrated in Fig. 4, with LRs for a direct match versus unrelated being well separated, with a few exceptions. Our findings indicate that samples with very low sequencing depth can still provide valuable information in, at least, one-to-one matches which is particularly relevant in cases where DNA results from crime scene traces are to be compared with a suspect’s or victim’s DNA. While we did observe some false positive comparisons, we found that by applying specific thresholds, based either on LR values or the number of SNPs used in the calculations, we could further reduce or eliminate these erroneous results (Fig. 4). Furthermore, while we mainly focused on evaluating the potential of the WGS analysis based on the Twist aDNA SNPs, for comparative reasons, the true potential of WGS should include analysis of all targets, i.e., the complete genome. We estimated this potential for one sample (019) and showed that more than 18 000 SNPs were called across 29 million target SNPs, which most likely is significantly informative in one-to-one matching in human identification cases. Furthermore, an alternative approach to increase the number of SNPs is genotype imputation. This approach is increasingly being investigated in both forensic genetics [73] and ancient DNA studies [74] and could serve as a valuable tool in our study to increase genotype calls, not only in absolute numbers but also for imputing forensically relevant SNPs. Further investigation into this approach could prove beneficial for similar datasets with incomplete SNP information in forensic and aDNA applications. While these results imply that WGS might not be optimal for routine forensic samples, it could provide valuable opportunities on a case-by-case basis, especially with those adaptations described in this study. Additional genetic information from WGS analysis could potentially be achieved with increased sequencing depth, however, it would be highly expensive and time-consuming, wherefore genotype imputation may be a powerful alternative. Moreover, WGS can provide additional information focusing on non-endogenous DNA which is not available from the targeted assays. This type of analysis have proved helpful in aDNA research for predicting the geographical origin of ancient samples [75] and obtaining information on individual health status [76], which could also prove valuable in a forensic context for specific cases.

A potential bias effect was observed within the Twist assay (Fig. 4B), as sample pairs with the highest LR was observed for Twist-Twist comparisons. The presence of allelic bias in capture-enriched strategies has been noted in previous studies [77, 78], and although specific design choices in the Twist assay were made to minimize this effect [58], the risk cannot be completely eliminated. In our dataset, we observed that such bias only had a practical impact when the number of compared SNPs was very limited. Importantly, by applying assay-specific thresholds, e.g. requiring at least 150 SNPs genotyped, only true matches were retained (Fig. 4B). This suggest that, in forensic applications, the influence of allelic bias is largely mitigated when sufficient SNPs are available for LR calculations, but becomes problematic in very low-data scenarios. For this reason, careful validation of capture-based SNP assays remains essential, with particular attention to define minimum SNP numbers required for reliable interpretation and identifying specific problematic SNPs that may be excluded from the panel.

As a consequence of the overall low call rate of the WGS data, and the potential bias effect in Twist, conclusions about the WGS genotype accuracy are limited. The genotype concordance rates illustrated in Fig. 5 should be interpreted with caution due to the overwhelming predominance of Y-chromosomal SNPs. The genotype concordance rate between FORCE and Twist (Fig. 5 and Additional file 1: Figure S7) was somewhat lower than expected for forensic applications, where high concordance is required for use in a legal setting. However, this represents only a few numbers of discordant genotypes. One efficient approach to reduce wrongful assignment of genotypes is replicate analysis, which we demonstrated in Fig. 5 for both FORCE and Twist data. Notably, genotype accuracy for the positive controls was high across all assays, indicating that the reduced concordance between FORCE and Twist likely stems from the challenging quality of the analyzed samples, suggesting improved performance for samples with increased quality or quantity. However, genotype errors should be considered since the proportion of errors will influence the correct classification of kinship [79]. In a study by Turner et al. [80], error rates of SNPs for FIGG applications between 1 and 5% resulted in unreliable results, underlining the need for minimizing genotyping errors. Similar concerns about the influence of error rates on accurate relationship inference have been raised by Watson et al. [81]. The selected FORCE thresholds in this study originated from [59] and internal validation, while the thresholds for the Twist and WGS data derived from [23]. Further evaluation and optimization of genotype calling thresholds could improve the results and reduce the risk of genotype errors. Thresholds should be determined by each laboratory based on internal validation prior to implementing any of the assays. Additionally, as shown herein, probabilistic genotyping could be applied as an alternative analytical step to determine genotypes, which also requires further validation prior implementation.

Our results demonstrate that SNP-based assays such as FORCE and Twist provide complementary and often more informative data compared to STRs, particularly when considering the absolute number of markers. While call rate alone did not differ significant between assays, this metric does not capture overall informativeness, as it ignores the assays target size. Assays with larger target panels, such as Twist and WGS, can provide increased SNP counts, which, in combination with probabilistic genotyping or genotype imputation, can significantly enhance the resolution and utility of the data. Thus, although STRs remain robust and reliable, our data shows that SNP assays offer a broader and more powerful source of information for forensic applications.

Not all evaluated assays are directly derived from the ancient DNA field, but our study illustrates how methodological advances from aDNA research can be adapted to forensic contexts. The capture-enrichment approach employed in Twist is a clear example of an aDNA-applied strategy successfully applied here, and WGS, although less common in forensics, has long been used in aDNA studies as a screening tool to assess DNA preservation. In addition, probabilistic genotyping from low coverage sequencing, represent a promising direction for forensic applications. More broadly, the use of large SNP panels reflects an aDNA inspired shift toward extracting maximal information from DNA samples, in contrast to the reliance on STR profiles. This study provides empirical evidence on the performance of such approaches in a forensic context, thereby supporting the ongoing transition toward implementing aDNA based assays in forensic genetics [14, 30, 52, 69].

We have demonstrated that all three SNP genotyping assays are powerful, and the choice of SNP assay should be guided by the forensic application and sample quality. For instance, we have previously shown that the FORCE panel is powerful for assigning up to fifth degree relatives [10], similar to other sparse forensic SNP panels such as the ForenSeq Kintelligence kit [11]. Identifying more distant relationships, which can be required in FIGG cases, may nevertheless benefit from denser SNP panels [82, 83]. Therefore, both the Twist and WGS assays could be better suited for such applications. Importantly, our results suggest that the Twist assay is particularly advantageous for highly degraded forensic samples. Although the overall call rates of FORCE and Twist were similar the substantially higher SNP target density of the Twist assay provides a greater opportunity to recover sufficient information from highly compromised DNA. This becomes especially powerful when combined with probabilistic genotyping which allows reliable interpretation even from sparse or imbalanced data. Thus, while FORCE may remain appropriate for kinship inference in cases where moderate SNP density suffices, Twist-like assays and WGS are preferable when maximal marker recovery is required.

It should be noted that the WGS libraries were sequenced across three independent runs, and the resulting FASTQ files were merged prior to genotype calling. Although this approach increases the total number of reads per sample, it does not necessarily provide an inherent advantage over the targeted assays. Sequencing depth per locus is influenced by several factors, including sequencing chemistry and kit capacity, the number of samples multiplexed within a run, and the total number of targets. These experimental design parameters not only influence sequencing depth but also have implications for cost and throughput, which are often among the most prioritized considerations for forensic laboratories. Each of the evaluated methods are more expensive than standard STR typing, however, the improvements in the outcome have been emphasized in this paper, and by several others [14, 15, 53]. Additionally, Budowle et al. [84] have made a comprehensive cost–benefit analysis of implementing large SNP panels and high throughput sequencing in a forensic context. They found that the societal benefits of implementing this type of technology are immense, and they provide convincing arguments for management of forensic laboratories to consider when discussing these types of technological implementations.

## Conclusions

This study demonstrates the potential of integrating ancient DNA methods into forensic genetics, providing a promising approach for analyzing challenging forensic DNA samples. While standard STR typing may prove insufficient for certain forensic applications, we explored three alternative SNP typing assays. Both the FORCE and Twist assay yielded increased genetic information, even from samples exhibiting exceptionally low amounts of DNA and severe degradation. Additionally, despite low sequencing depth, a probabilistic genotyping approach applied to the WGS data proved powerful for one-to-one matching, underscoring the utility of WGS even under challenging conditions. Further investigation is recommended to assess genotype accuracy for low-quality and low-quantity samples, with a focus on internal validation to establish reliable SNP calling thresholds and reduce genotyping errors. Overall, our findings support the adoption of these SNP typing assays into forensic genetics to strengthen analysis capabilities and improve outcomes in complex forensic cases.

## Methods

### Workflow overview

A schematic overview of the methodology is illustrated in Fig. 1. We have compared standard STR typing with three different SNP genotyping assays: 1) a PCR enrichment targeted forensic SNP panel, the FORCE QIAseq assay (Qiagen, Hilden, Germany) [59], 2) a hybridization capture-based target enrichment panel, the Twist human aDNA panel (Twist Biosciences, San Francisco, CA, USA) [58] and 3) whole-genome sequencing (WGS). For all assays, the same DNA extract solution was used, and genotype comparison was performed for the three different SNP genotyping assays. Methodological details are provided in the following sections.

### Sample selection

All samples were handled and analyzed in accordance with ethical approval by the Swedish Ethical Review Authority (Dnr 2022–05395-01). Twenty skeletal bone and tooth samples from authentic unidentified missing person cases, received by the National Board of Forensic Medicine in Linköping, Sweden were selected. All samples were expected to be of modern origin, i.e., less than 30 years old. Table 1 specifies the type of bone material selected for the study. Two samples (NA12877 and NA12878) provided by NIGMS Human Genetic Cell Repository at the Coriell Institute for Medical Research (Camden, NJ, USA) were used as positive controls in all subsequent analysis, except for DNA extraction, as they were provided as extracts.

### Sample preparation, DNA extraction and quantification

All samples were decontaminated via UV irradiation (254 nm) at approximately 1 J/cm^2^ per side. The outer surface of the samples was then drilled to remove remaining contaminants. The samples were then further drilled to access the inner bone material and approximately 50 mg bone powder was acquired for subsequent DNA extraction. The DNA extraction was performed according to the standard protocol for aDNA extraction at the Centre for Palaeogenetics, Stockholm University, described in detail in [43, 85]. The samples were divided into three different extraction batches and one extraction blank per batch was used. An in-house extraction buffer was prepared by mixing 6.3 mL 8 M urea, 40.7 mL 0.5 M EDTA (pH 8.0), and 3 mL 10% SDS, resulting in a final concentration of approximately 1 M urea, 0.4 M EDTA, and 0.02 M SDS. One milliliter extraction buffer and 100 µg Proteinase K was added to each sample, and the solutions were incubated in a hybridization oven at 37° C for 48 h. SDS is not included in the standard protocol but was chosen since we expected higher fat content in these more modern samples compared to ancient samples. The supernatant was transferred to Amicon Ultra-4 columns (30 kDa cut off, Merck Millipore, Darmstadt, Germany) and centrifuged at 4000 rcf for ca 10 min until ca 100 µl concentrate was obtained. Following, the concentrated supernatant was purified with the MinElute purification Kit (Qiagen) and eluted in 70 µl EB buffer (Qiagen). The DNA extracts were quantified with Qubit 2.0 fluorometer (Thermo Fisher Scientific, Waltham, MA, USA). A second quantification approach was performed with the PowerQuant System (Promega Corporation, Madison, WI, USA) on the ABI 7500 Real-Time PCR System (Thermo Fisher Scientific), following the manufacturer protocol. Eight samples (001, 002, 003, 007, 008, 010, 012, and 013) were selected for re-extraction since they displayed insufficient DNA retrieval, i.e., less than 1% human sequences from the initial WGS analysis. The re-extraction was done with two separate extraction protocols, the Dabney extraction [86] and Ecogen extraction [87]. In the Dabney protocol, the DNA binding buffer was slightly modified by adding the following to 500ml Qiagen PB buffer: 500 µl Phenol red (1000x, Sigma P0290), 15 ml 3 M NaAcetate pH 5.2 (Sigma S7899), 1.25 ml NaCl (5 M) and adjusted with 37% HCl to pH 5 if needed. After the incubation period, 20 ml of this buffer was combined with 1 ml of the digestion buffer for subsequent DNA binding. Only WGS analysis was performed with these re-extracted DNA samples and the quantification results displayed in Table 1 refers to the initial extraction of all samples.

### STR analysis

Nineteen of the samples, the three extraction blanks and the two positive controls were assessed by STR DNA typing analysis. This was performed using the PowerPlex Fusion 6C System (Promega Corporation) with amplification according to the manufacturer protocol and using a HID Veriti Thermal Cycler (Thermo Fisher Scientific), ABI 3500xL Genetic Analyzer (36 cm capillary array; POP4; injection parameters: 24 s/1.2 kV; analytical thresholds: blue channel 95 rfu, green 140 rfu, yellow 85 rfu, red 135 rfu and purple 95 rfu), 3500 Series Data Collection Software v4.0.1 and GeneMapper ID-X 1.6 (Thermo Fisher Scientific). Twenty-three autosomal STRs, three Y-STR markers and Amelogenin were targeted with the kit. The recommended DNA input was 1 ng with a maximum template volume of 15 µl, but due to the limited volume of DNA extract, less than 1 ng was used for all except one sample (011), see Additional file 2: Table S1 for all DNA input amounts in the amplification.

### Library preparation of FORCE QIAseq

Library preparation of the FORCE SNPs was done in accordance with the QIAseq Targeted DNA panel protocol (Qiagen) [88]. The maximum DNA volume (16.75 µl) was used for all samples except for the positive controls, where 20 ng DNA was used. The first step of the library preparation was a multienzymatic reaction including fragmentation, end-repair and A-addition performed at 32 °C for 24 min and at 72 °C for 30 min. Thereafter, adapters were ligated at room temperature and cleaned twice with QIAseq magnetic beads. Target enrichment was performed with six cycles of amplification and purified according to the protocol. A universal PCR with 19 cycles was performed and followed by two additional rounds of clean-up to reduce the amount of adapter dimers [59]. The resulting DNA libraries were quantified using the Qubit 2.0 fluorometer and the DNA integrity was checked using the 2100 Bioanalyzer (Agilent Technologies, Santa Clara, CA, USA) (data not shown). DNA libraries were denatured and diluted to 10 pM, and three to four samples were pooled in equimolar concentrations and sequenced together on a MiSeq FGx (Qiagen) for paired-end 151 by 151 bp sequencing.

### Whole-genome library preparation

DNA libraries were prepared following the Meyer and Kircher protocol [89] but in contrast to the protocol, we applied unique dual indexing. The library preparation was conducted utilizing two different approaches: one involving an initial enzymatic fragmentation step, and the other without any fragmentation. The enzymatic shredding was done with NEB fragmentase and not by sonication as described in the protocol [89]. Libraries for the positive controls (NA12877 and NA12878) were only built by including the fragmentation step. The remaining steps of the procedure followed the protocol. Briefly, blunt-end repair was conducted for all samples to ensure uniform and intact DNA fragments, followed by a magnetic bead-based clean up and adapter ligation. The number of amplification cycles was determined by qPCR analysis of the libraries. Subsequently, the libraries were amplified with PCR and purified with magnetic beads. The final libraries were analyzed on a 2100 Bioanalyzer with the High Sensitivity DNA kit (data not shown). Another set of libraries were prepared from the above libraries but amplified with three additional PCR cycles which were subsequently analyzed solely with WGS.

### Target enrichment via hybridization capture

Hybridization capture-based target enrichment followed the procedures described in the Twist Target Enrichment protocol (Twist Bioscience) [90], with the modifications outlined in [58] to address the Twist human ancient DNA panel (Twist Bioscience). The protocol was based on a singleplex setup and one round of hybridization capture. The recommended input amount of the above prepared amplified and indexed whole-genome DNA library was 1 µg. However, the amount of the DNA libraries built above was less than 1 µg from the initial amplification round. Therefore, a second amplification of the DNA libraries was performed with the following PCR program: initial denaturation at 98 °C for 2 min, 14 cycles of 98 °C for 20 s, 60 °C for 30 s and 72 °C for 45 s, following a final extension at 72 °C for 5 min. The amplified DNA libraries were purified according to [89]. From this extra round of amplification, 1 µg of the DNA libraries were dried with a magnetic bead-based assay, as described in the Appendix of the protocol [90]. Universal blockers and blocker solution were added to the dried libraries and incubated at 95 °C for 5 min in a thermal cycler with heated lid at 105 °C, then brought to room temperature for 5 min. A probe solution consisting of Hybridization Mix, the Twist custom ancient DNA probes and water was incubated at 95 °C for 2 min in a thermal cycler with heated lid at 105 °C, then cooled on ice for 5 min and incubated at room temperature for 5 min. The probe solution and hybridization enhancer solution were added to the tube with libraries and incubated at 62 °C for 16 h in a thermal cycler with the lid at 65 °C for hybridization. Streptavidin binding beads were used to bind the hybridized targets at room temperature for 30 min. One wash of the beads was performed with Wash Buffer 1 followed by three stringent washes at 49 °C with Wash Buffer 2. The washed and bound streptavidin beads were eluted in water and half of the elution volume (22.5 µl) was used for amplification. The Equinox Library Amp Mix (2x) and amplification primer (ILMN) were added and PCR amplification with 23 cycles was conducted. A 1.8X magnetic bead based clean up followed the amplification and 30 µl purified and enriched library was eluted in nuclease free water. Qubit 2.0 fluorometer was used to quantify the enriched libraries. The fragment length distribution of the libraries was assessed with 5200 Fragment Analyzer and the HS NGS Fragment kit (Agilent Technologies) (data not shown). All samples were diluted based on the quantification measurement and the average fragment length distribution and pooled in equimolar concentrations to a final concentration of 10 nM. Four pools, with eight to nine samples each, were loaded onto four separate lanes, with each pool being assigned to a distinct lane on a NovaSeq X Plus 25B-300 flow cell for paired-end 151 by 151 bp sequencing.

### Whole-genome sequencing

Whole-genome sequencing was performed in three separate sequencing runs, all using the NovaSeq X Plus platform with a 151 by 151 bp setup utilizing the 10B mode flow cell. From this setup, the theoretical average coverage per SNP and sample is 26x, assuming ideal conditions with no PCR duplicates or other sequencing biases that could reduce the coverage. The first run included all libraries from the initial PCR amplification and were distributed on three separate lanes. A second sequencing run was done to increase the sequencing read depth. Samples that displayed more than 1% human proportion from the first sequencing were re-sequenced in two separate lanes. Additionally, a third lane was used to sequence all libraries with three additional PCR cycles. A final sequencing run was performed with the eight successfully re-extracted samples which were sequenced on 0.5 lane.

### Bioinformatic analysis

FASTQ files from each of the three SNP genotyping assays were bioinformatically analyzed in CLC Genomics Workbench V. 23.0.4 (Qiagen) [91]. Individual FASTQ files from the FORCE and Twist assays were imported to CLC. FASTQ files from all lanes across the three WGS sequencing runs were merged prior to import into CLC. The final dataset thus included all WGS libraries, including fragmented and non-fragmented libraries, libraries with additional PCR cycles, and re-extracted libraries. The bioinformatic analysis of the FORCE QIAseq assay followed the workflow described in [59]. Additional file 1: Figure S3 illustrates an overview of the FORCE workflow in CLC. Default settings were applied for all parameters in CLC except for the *Map reads to Reference* tool where the *Length fraction* was set to 0.8, the *Similarity fraction* to 0.95 and *Global alignment* to True. Detailed descriptions of each parameter is described in [91].

The sequencing reads from Twist was processed in CLC by trimming the reads from adapters with the *Trim Reads* tool. Thereafter, human reads were filtered with the *Taxonomic profiling* tool included in the Microbial Genomics Module in CLC. The taxonomic profiling tool in combination with the read mapping performs computationally substantially faster than solely using the read mapping tool, wherefore this approach was applied. However, this comes with the drawback that some human reads are, possibly, discarded by the taxonomic profiling tool. Briefly, this tool scans for and extracts reads according to a reference genome (human in this case). Thereafter, the reads were mapped to human reference hg19 with *Map Reads to Reference* tool. PCR duplicate reads were removed with *Remove Duplicate Mapped Reads* and, lastly, *Identify Known Mutations from Mapping* was performed, which counts the number of reads of each nucleotide for the target regions. In addition, the tool outputs the forward/reverse read balance and the average Phred score for each nucleotide. Default settings were applied for all parameters in CLC. The WGS data followed the same CLC workflow as Twist with the addition of a *Local realignment* step following the removal of duplicate reads. An overview of the workflows is presented in Additional file 1: Figures S4 and S8.

Final genotype calling was performed with an in-house developed R-script (R version 4.3.3) [92] for both the Twist and the WGS data. Specific thresholds were applied for the coverage, allelic balance, Q-score, and forward/reverse read ratio (Additional file 2: Table S5). For the FORCE data, genotype calling was done in Microsoft Excel based on specific thresholds (Additional file 2: Table S3). While internally validated genotype calling thresholds were used for FORCE, thresholds for Twist and WGS were derived from previous studies [23]. A multivariate linear regression analysis was conducted in R version 4.3.3 [92] to identify and compare the influence of different parameters on the number of called SNPs. The following parameters were included: DNA input concentration, total sequences, trimmed sequences, human reads filtered with the *Taxonomic profiling* tool, reads mapping to the human reference genome, reads mapping on-target, and PCR duplicates.

### Genotype accuracy and concordance

Genotype accuracy for each of the three SNP genotyping assays was assessed by comparing observed genotypes for the two positive controls (NA12877 and NA12878) with genotypes obtained from previously published high-depth FASTQ files [93] analyzed with the CLC workflow presented in Additional file 1: Figure S8 and typing thresholds according to Additional file 2: Table S5. Genotype concordance was assessed between FORCE and Twist data for nine samples (all samples that had one or more overlapping SNPs typed in both assays). Genotype concordance for fifteen samples (including the two positive controls) was done between the typed genotypes for Twist and WGS. Additionally, one sample was analyzed in duplicate with FORCE, wherefore concordance analysis within FORCE was performed for that sample. Similarly, four samples were analyzed in duplicate with Twist and within method concordance between those samples was performed.

Due to low coverage data, genotype concordance between the Twist and WGS data was further assessed using a probabilistic genotyping approach. Mostad et al. [72] described a likelihood-based model where, in contrast to for instance ngsRelate [94], genetic linkage is accounted for. We used the implementation in FamLink2 [60] where a likelihood ratio (LR) is calculated comparing the genetic data under the two competing hypotheses, H1: The two samples originates from the same donor and, H2: The samples are from two different donors. Fifty thousand autosomal SNPs were selected for the LR calculations based on: 1) availability of reference data, derived from the 1000 Genomes Project [95], and 2) the SNPs with highest read count across all samples. Pairwise comparisons of H1 versus H2 for all samples including the two positive controls were performed. The following software specific settings were applied in FamLink2: *Genotype Likelihoods* was activated, *Sequencing/mapping error* was set to 0.01 and *Sample specific error* was set to 2.

### Assay comparison

The performance of the examined assays (STR, FORCE, Twist and WGS) was evaluated based on the call rate per sample for each assay, which was calculated by dividing the number of observed markers with the total number of markers in each assay. A one-way ANOVA test was conducted in R version 4.3.3 [92] to determine if there was statistically significant difference in the mean call rates of the four assays. The following hypotheses were tested: Null hypothesis *– All assays have the same average call rate*, versus the Alternative hypothesis *– At least one assay has a different average call rate*. Following, a post-hoc Tukey's HSD test was performed in R version 4.3.3 [92] for each pair of assays to determine which assay that differs from the others. Additionally, due to the overall low coverage from the WGS data, comparisons were also done based on a dynamic SNP calling approach. The top 5000 SNPs with highest coverage were selected for each sample and the resulting call rate, based on 5000 SNPs as maximum and the same typing thresholds as described in Additional file 2: Table S5, was used for the statistical evaluation.

## Supplementary Information

Additional file 1: Figure S1. Insert size of mapped reads, Figure S2. Damage plots, Figure S3. CLC workflow for FORCE, Figure S4. CLC workflow for Twist, Figure S5. Genotype concordance between FORCE and Twist, Figure S6. Genotype concordance between Twist and WGS, Figure S7. Genotype concordance excluding Y-SNPs, Figure S8. CLC workflow for WGS

Additional file 2: Table S1. DNA amount for STR analysis, Table S2. FORCE SNPs, Table S3. Force genotyping thresholds, Table S4. Sequencing metrics FORCE, Table S5. Twist and WGS genotyping thresholds, Table S6. Sequencing metrics Twist and WGS, Table S7. Discordant SNPs, Table S8. *p*-values multivariate linear regression analysis, Table S9. *p*-values assay comparison.

## Data Availability

The data that support the findings of this study are not openly available due to reasons of sensitivity. Access may be considered upon reasonable request to the National Board of Forensic Medicine in Sweden (rmv@rmv.se) (mailto:rmv@rmv.se). Each request is evaluated on a case-by-case basis to ensure compliance with applicable legislation, including assessment of the proposed research question, the presence of appropriate ethical approval, and other relevant considerations, and will be addressed in a timely manner. The in-house developed R-script for final genotype calling of the Twist and the WGS data is available in a public GitHub repository and in Zenodo under the MIT license [96, 97].
