# Supplementary material for: Comparative assessment of SNP genotyping assays for challenging forensic samples utilizing ancient DNA methods
Source: Genome Biol. 2025 Dec 23;26:433. doi: 10.1186/s13059-025-03912-z (PMC12723910; doi:10.1186/s13059-025-03912-z)
Supplement: Supplementary file 1 — Additional file 1: Figure S1. Insert size of mapped reads, Figure S2. Damage plots, Figure S3. CLC workflow for FORCE, Figure S4. CLC workflow for Twist, Figure S5. Genotype concordance between FORCE and Twist, Figure S6. Genotype concordance between Twist and WGS, Figure S7. Genotype concordance excluding Y-SNPs, Figure S8. CLC workflow for WGS [file 13059_2025_3912_MOESM1_ESM.docx]

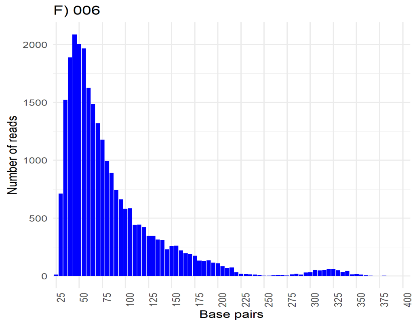

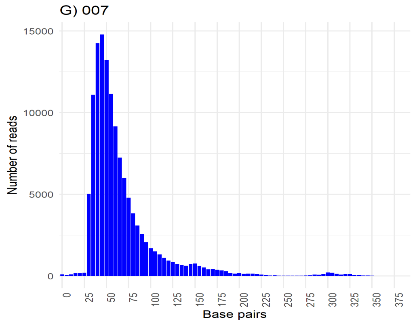

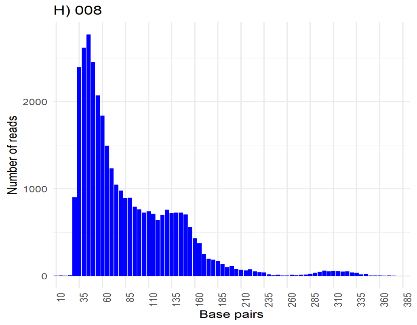

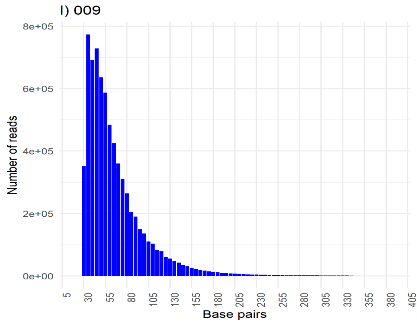

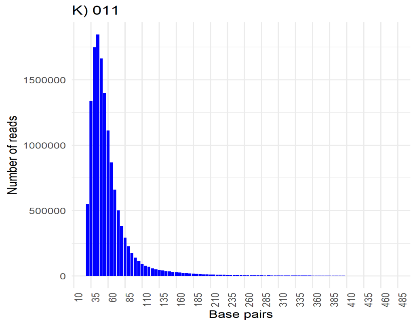

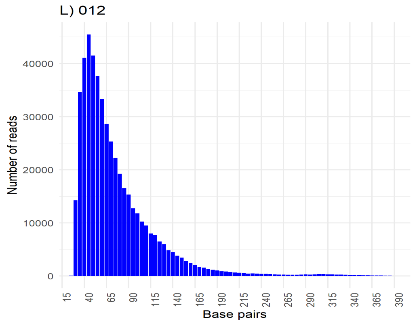

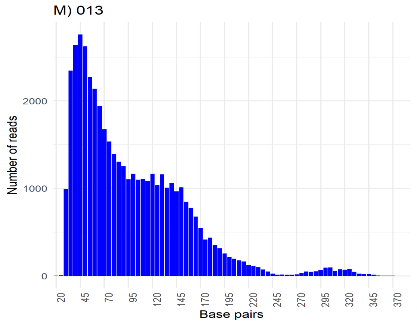

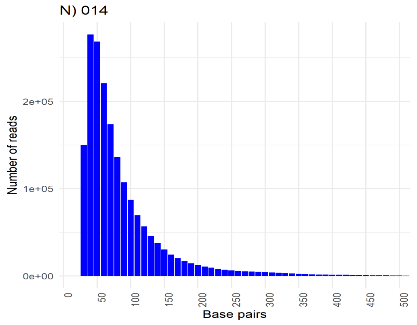

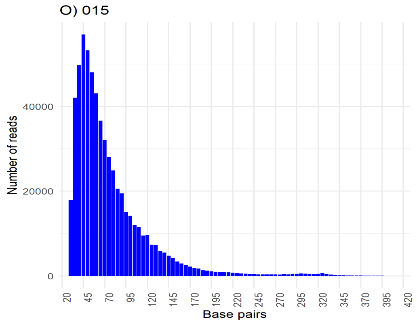

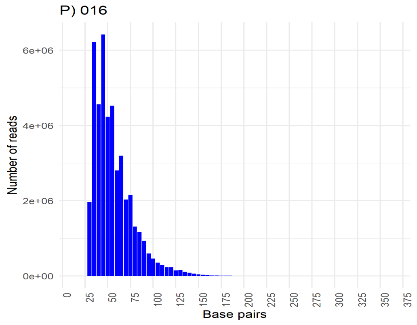

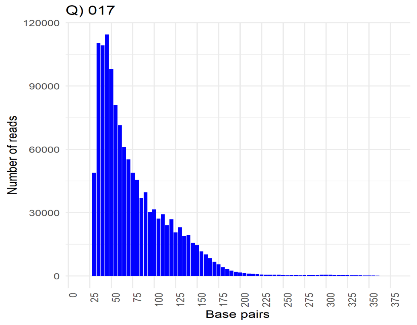

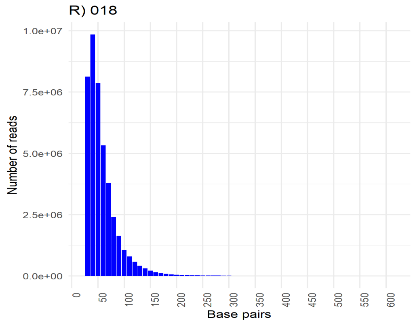

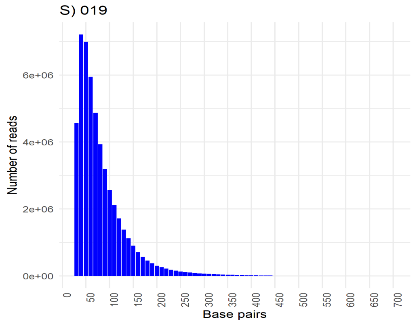

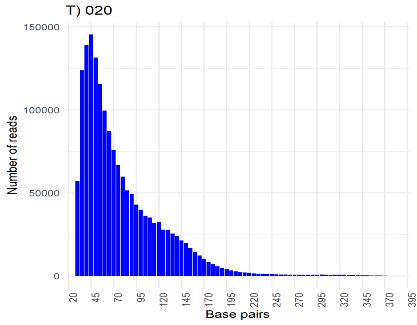

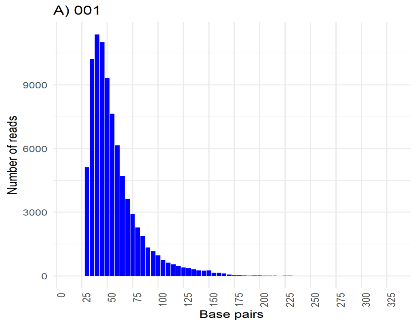

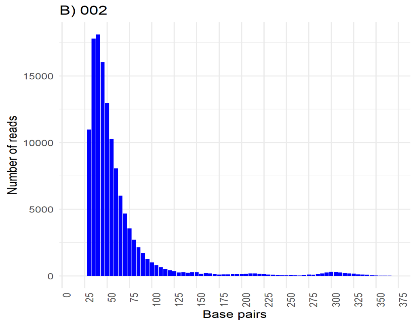

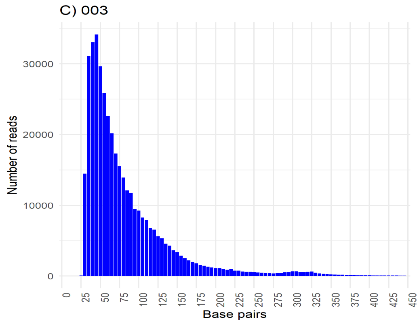

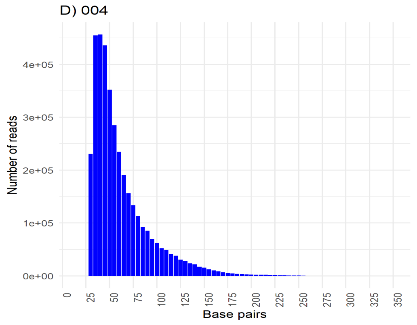

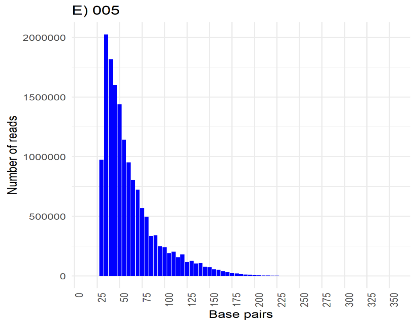

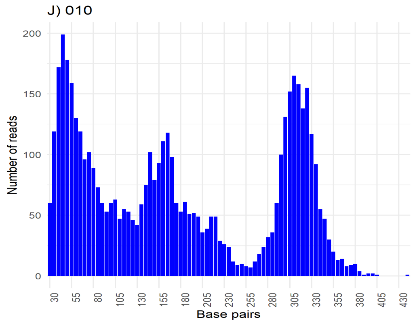


**Figure S1:** Insert size of mapped reads for all samples.


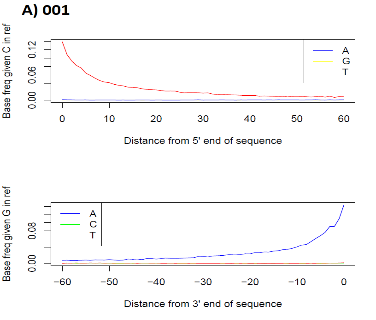

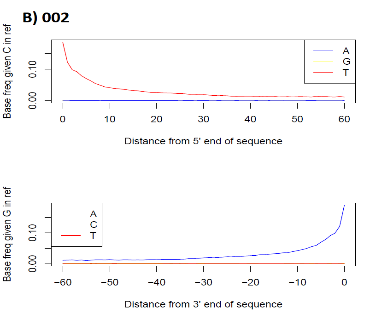

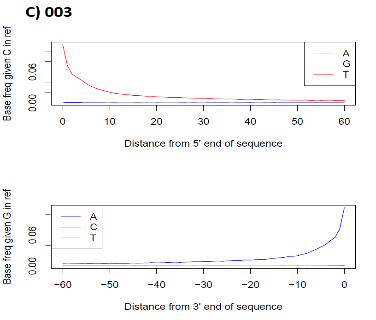

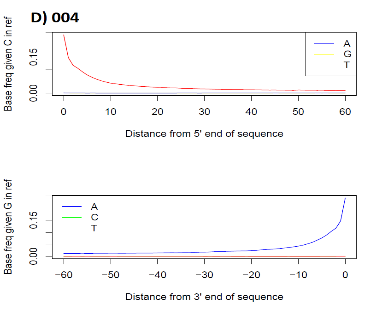

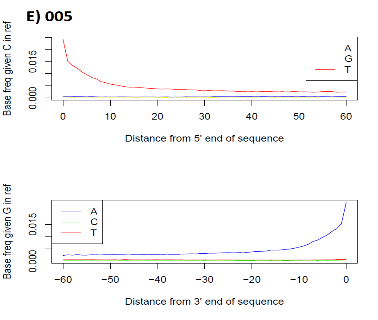

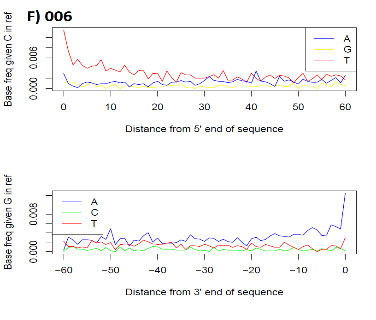

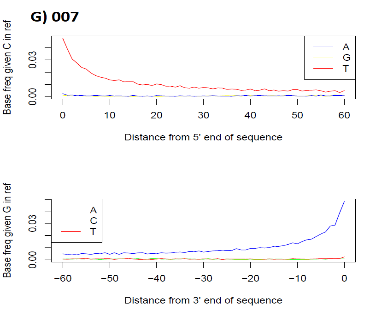

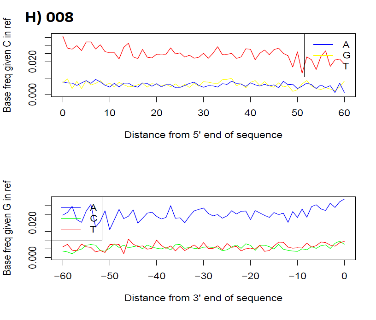

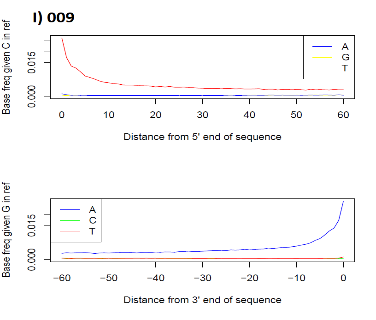

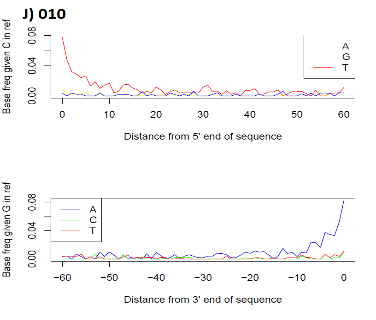

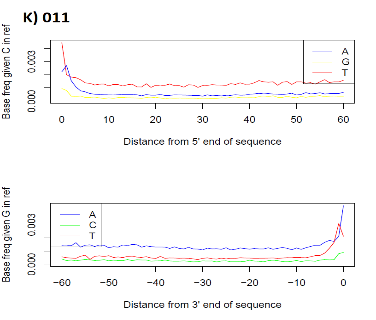

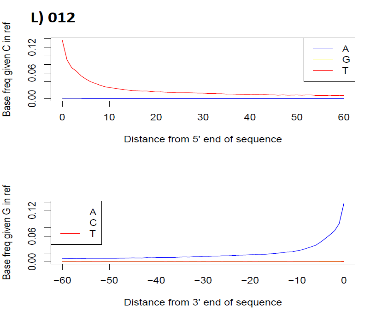

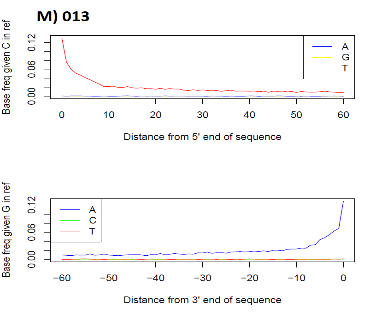

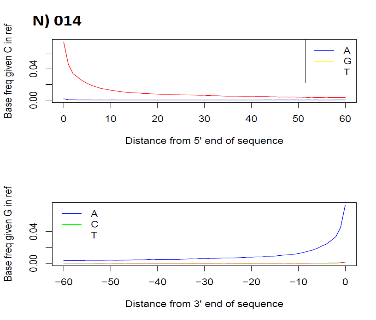

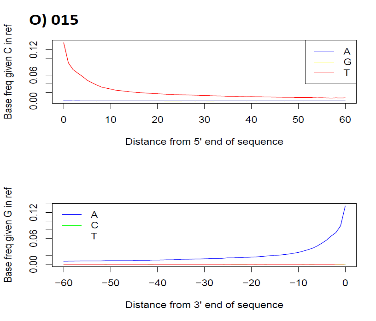

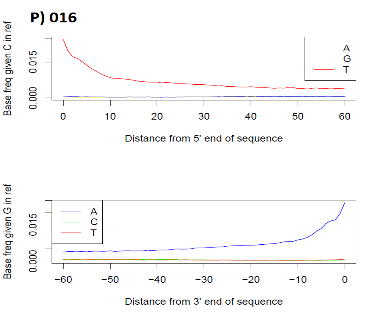

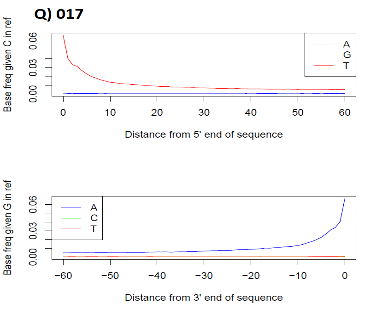

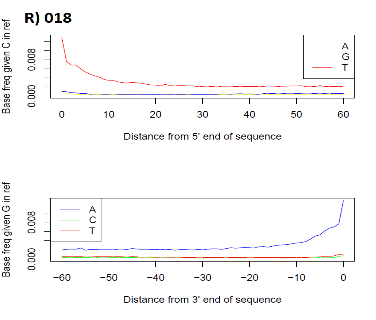

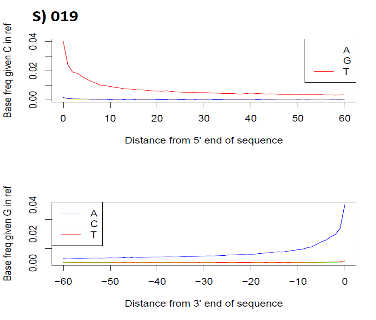

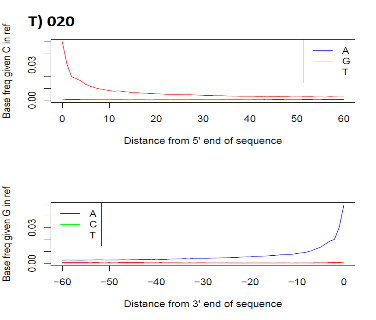


**Figure S2:** Damage plots for all samples.


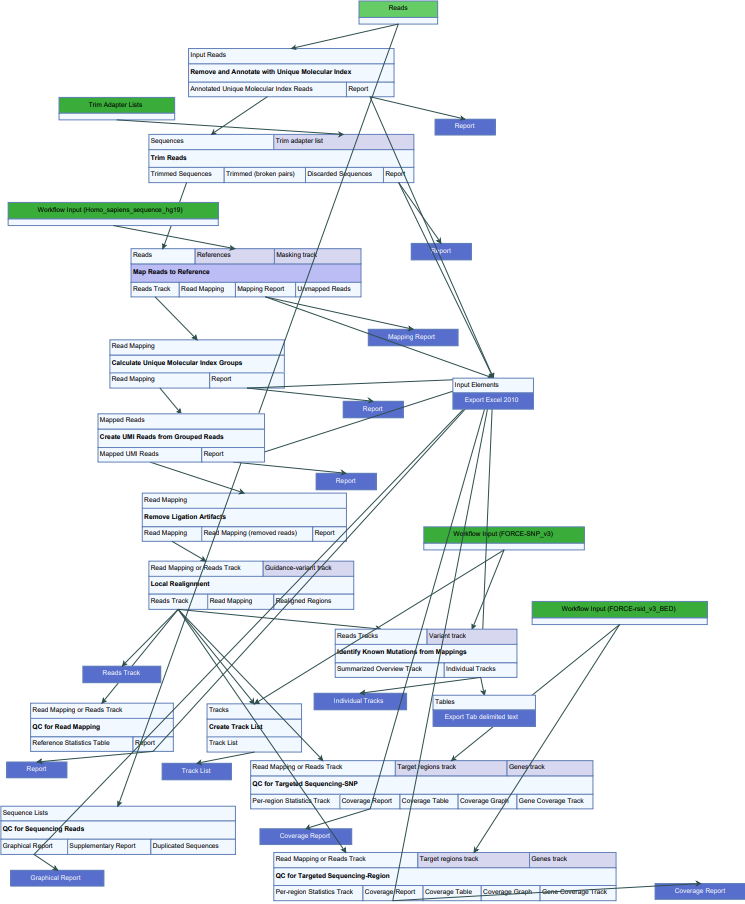


**Figure S3:** An overview of the workflow in CLC Genomics Workbench for the FORCE assay.


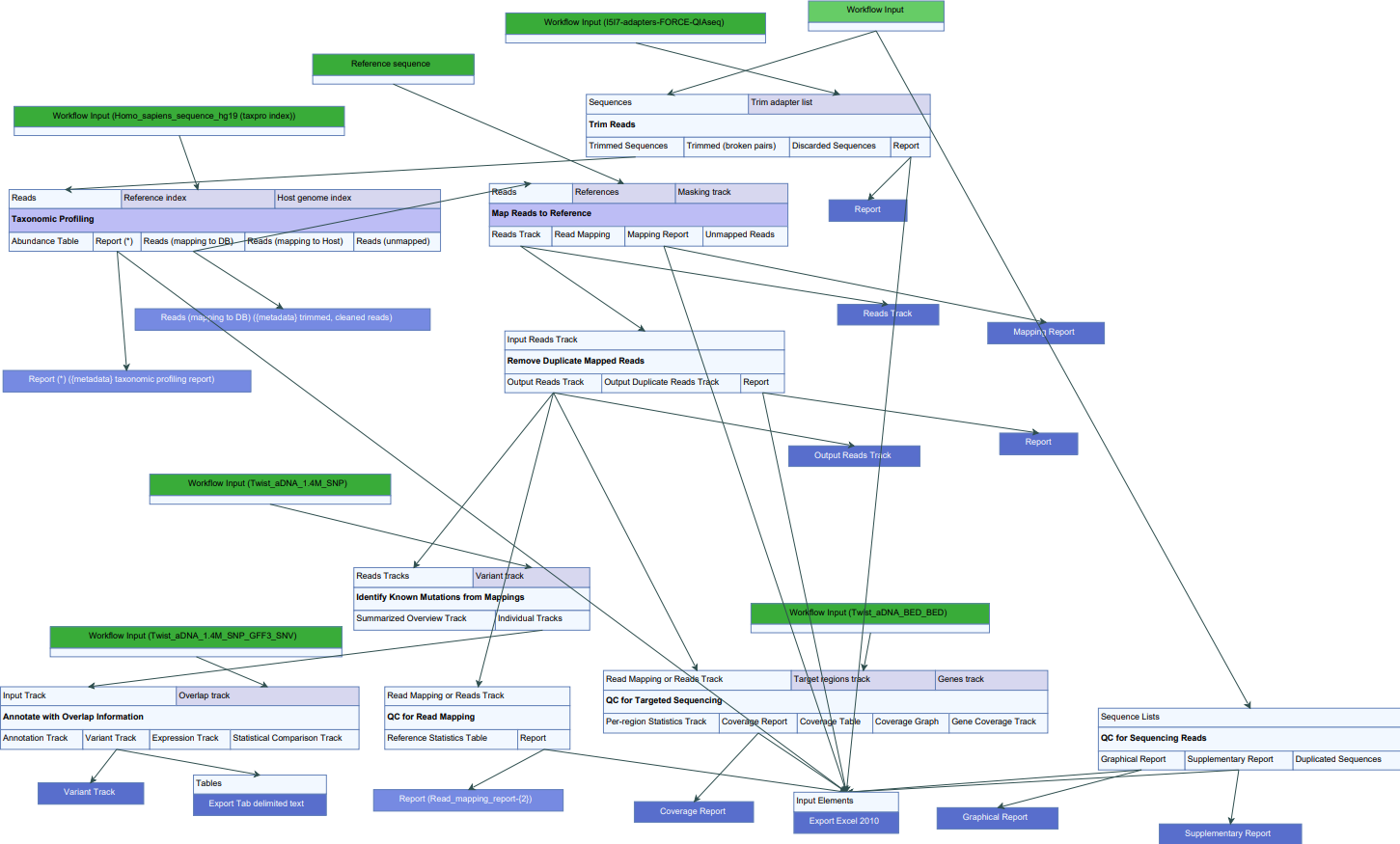


**Figure S4:** An overview of the workflow in CLC Genomics Workbench for the Twist assay.

**Figure S5:** **A)** Genotype concordance between all sample pairs with overlapping typed SNPs between FORCE and Twist. **B)** The number of overlapping typed SNPs between FORCE and Twist.

**Figure S6:** **A)** Genotype concordance between all sample pairs with overlapping typed SNPs between Twist and WGS. **B)** The number of overlapping typed SNPs between Twist and WGS.


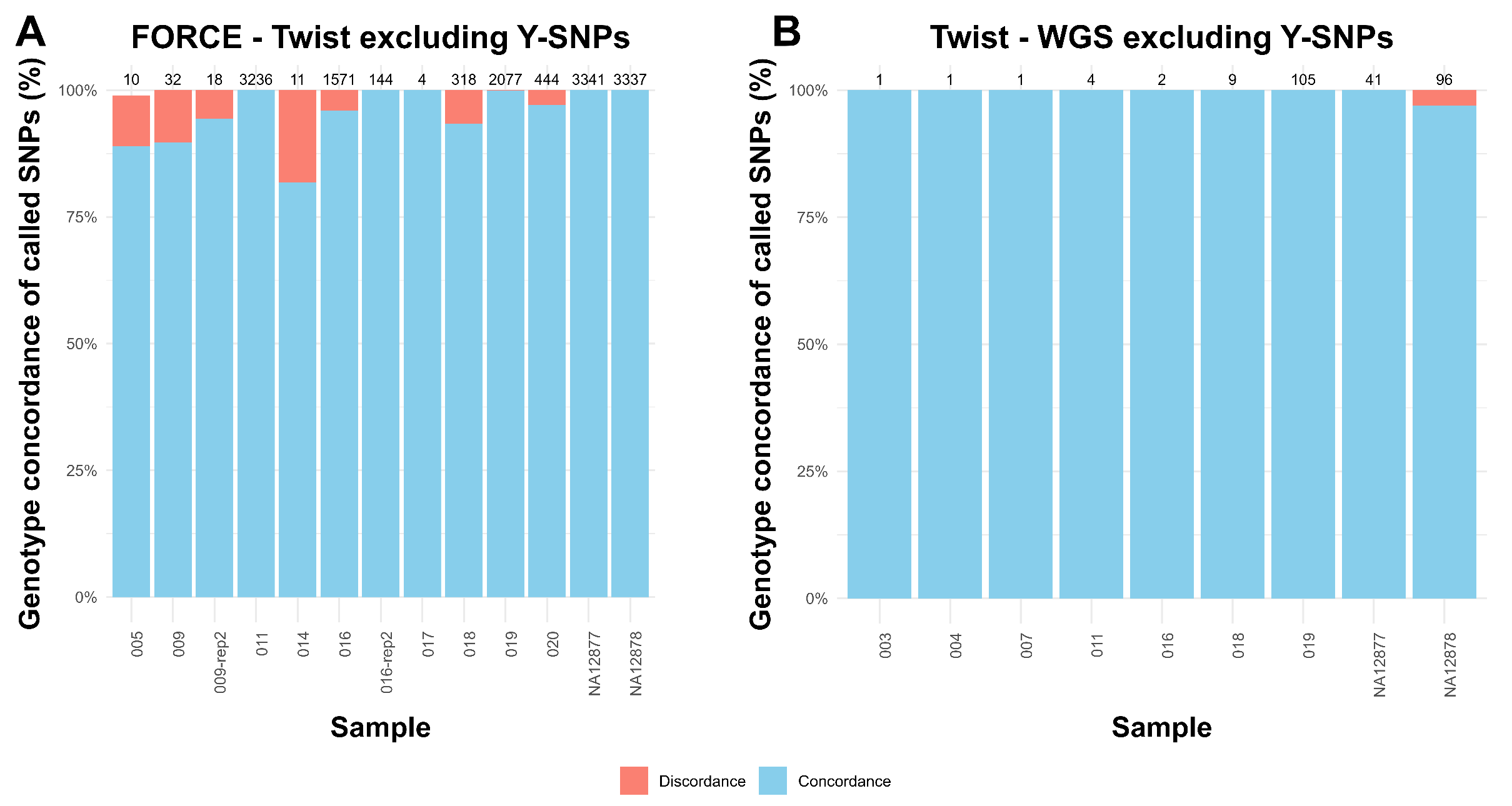


**Figure S7:** Genotype concordance (blue) and discordance (red) for samples with overlapping typed genotypes, excluding Y-SNPs. The number above each bar represents the number of overlapping typed SNPs in the comparison. Sample 009 was analyzed in replicate with the Twist assay, labeled as 009-rep2 in the figure. Sample 016 was analyzed in replicate with the FORCE assay, labeled as 016-rep2. **A)** shows concordance between FORCE and Twist, and **B)** represents the Twist and WGS comparisons.


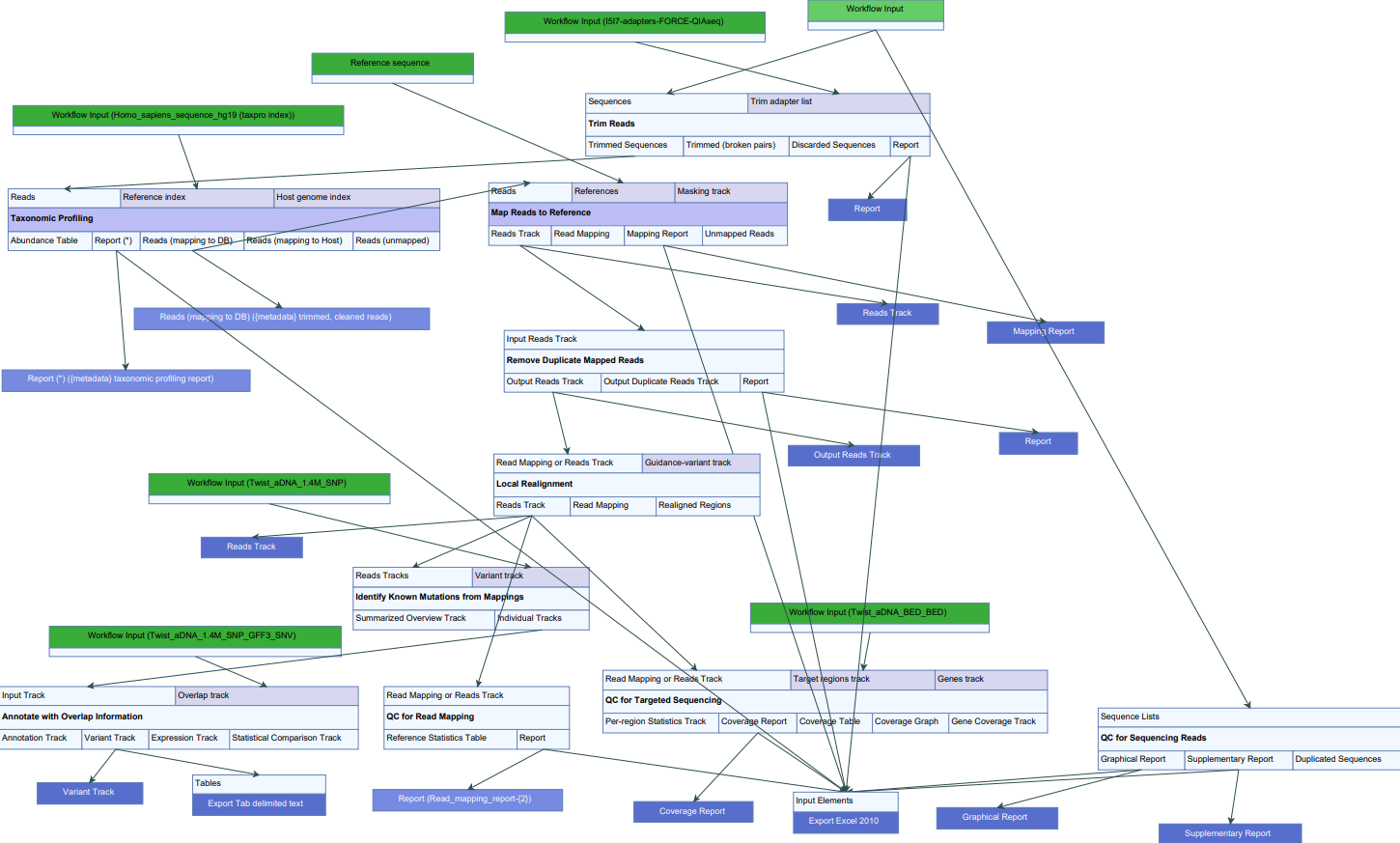


**Figure S8:** An overview of the workflow in CLC Genomics Workbench for the WGS assay.
